# Exact solutions and instability analysis of a fifth-order nonlinear evolution equation exhibiting multi-scale wave structures

**DOI:** 10.1038/s41598-026-60986-w

**Published:** 2026-07-13

**Authors:** Wafy M. Hasan, Hamdy M. Ahmed, Ahmed M. Ahmed, Haytham M. Rezk, Wafaa B. Rabie

**Affiliations:** 1 Department of Mathematics, Faculty of Science, Al-Azhar University, Cairo, Egypt; 2 Department of Basic Sciences, Faculty of Engineering Technology, ElSewedy University of Technology, Cairo, Egypt; 3 Department of Physics and Engineering Mathematics, Higher Institute of Engineering, El Shorouk Academy, Cairo, Egypt; 4 Department of Mathematics, Faculty of Science, Luxor University, Taiba, Luxor, Egypt; 5 Luxor National University, Faculty of Computing, Information Technology and Artificial Intelligence, Luxor, Egypt

**Keywords:** Fifth-order nonlinear equation, Exact solutions, Nonlinear waves, Stability analysis, Soliton dynamics., Mathematics and computing, Physics

## Abstract

A fifth-order nonlinear partial differential equation is analyzed to construct exact traveling wave solutions through the modified extended mapping method. By converting the governing equation into an equivalent ordinary differential form, the approach enables the derivation of multiple classes of analytical solutions in a unified manner. These solutions include localized structures such as bright and dark solitons, singular waveforms, periodic and singular periodic patterns, as well as rational, exponential, and elliptic function solutions represented by both Weierstrass and Jacobi formulations, in addition to combined bright–dark soliton structures. To further characterize the system, a linear perturbation framework is applied to assess stability properties. The resulting dispersion relation is examined to determine parameter regimes associated with the onset of instability and the evolution of disturbed wave states. This work provides a generalized analytical perspective for exploring complex nonlinear wave behavior in higher-order systems and offers a foundation for future studies involving multi-scale wave interactions and stability features.

## Introduction

Partial differential equations (PDEs) serve as cornerstone mathematical models in numerous scientific and engineering fields, enabling the description of phenomena that evolve in both space and time. They are especially vital for capturing wave propagation, fluid flow, and other processes in which nonlinearity generates intricate structures such as shocks, turbulent states, and solitary waves. For instance, the nonlinear Navier–Stokes equations remain the primary tool for analyzing complicated fluid motions, including turbulence, vortex formation, and wave-breaking events^1,2^. Likewise, the Korteweg–de Vries (KdV) equation has played a central role in elucidating the nature of nonlinear wave propagation and the emergence of solitons in shallow-water systems^3,4^. The utility of PDEs extends far beyond classical physics: in environmental and geophysical contexts, they are routinely employed to model atmospheric and oceanic wave dynamics, while nonlinear advection–diffusion equations are used to track pollutant transport in air and water bodies^5,6^. Engineering disciplines also rely heavily on PDEs for studying shock-wave phenomena, optical pulse evolution in fibers^7,8^, climate variability, and extreme meteorological events^9,10^. The extraordinary breadth of these applications highlights the indispensable role of PDEs in tackling real-world problems across diverse domains. Exact solutions to nonlinear evolution equations (NLEEs) can be constructed through a rich array of analytical techniques. Notable examples include the extended sinh–Gordon equation expansion method, which exploits known solutions of the sinh–Gordon equation to generate new solutions for wider classes of NLEEs^11^; the modified extended tanh-function method, which enhances the classical tanh expansion to yield richer solution families^12,13^; the modified extended direct algebraic approach, offering a structured framework that converts complex NLEEs into solvable algebraic systems^14,15^; and the modified extended mapping method, which establishes versatile functional mappings to interconnect different solution types and thereby broaden the scope of treatable equations^16,17^. Additional powerful tools encompass the modified Sardar sub-equation technique^18^, the extended F-expansion method^19,20^, the improved modified extended tanh-function scheme^21,22^, the homogeneous balance method^23^, Bäcklund transformations, and Riccati–Bernoulli sub-equation approaches^24^, among others. Collectively, these methodologies provide essential instruments for exploring sophisticated physical processes, including wave propagation, fluid instabilities, and heat transfer phenomena, while bridging rigorous theoretical developments with practical applications in mathematics, physics, and engineering. Investigating NLEEs in higher-dimensional settings continues to be a dynamic and critical area of research, owing to their capacity to represent intricate phenomena arising in fluid mechanics, nonlinear optics, plasma physics, and environmental modeling. The motivation for this study stems from the significant challenges inherent in analyzing high-order nonlinear partial differential equations (NPDEs). Specifically, we focus on the recently introduced $(1+1)$-dimensional Wazwaz fifth-order equation, expressed as^25^:

1 $$\begin{aligned} u_{t} + u_{xxt} + \mu u_{x} - u u_{xxx} - u u_{x} - 3 u_{x} u_{xx} - \mu u_{xxxxx} = 0, \end{aligned}$$

where $u = u(x, t)$ is a real function, and $\mu$ is a constant parameter influencing the dispersive and nonlinear properties. In the study by Wazwaz^25^, a new Painlevé integrable fifth-order equation is introduced, with its integrability confirmed through the Painlevé test to examine compatibility conditions via Laurent series expansion, leading-order analysis, and resonance points (found at −1, 4, 5, 6). Employing the simplified Hirota bilinear method, along with dispersion relations and phase shifts, the work derives multiple soliton solutions, including single solitons, two-soliton, and three-soliton solutions. Additional exact solutions feature distinct physical structures, such as kink solutions via the tanh method and coth method, and periodic solutions via the tan method, cot method, and cosine method. This equation extends classical KdV-type models by incorporating higher-order dispersive and nonlinear terms, offering enhanced insights into solitary wave dynamics, stability, interactions, and dissipation in fields like fluid mechanics and nonlinear optics. Graphical analyses further depict the spatiotemporal behavior of these waves under different parameters. The Painlevé integrability of Eq. (1), established in^25^ via the Painlevé test, guarantees that the equation possesses the Painlevé property – solutions have no movable branch points, only movable poles. This property lends confidence to the existence of a rich solution space and is consistent with complete integrability in the sense of possessing a Lax pair, infinite conservation laws, and multi-soliton solutions. However, we emphasize that the MEMM employed in this work is a direct algebraic method that does not explicitly exploit the integrable structure (such as inverse scattering transform, Bäcklund transformations, or Darboux transformations). Rather, the MEMM operates at the level of direct substitution and algebraic manipulation to systematically generate traveling-wave solutions. The Painlevé integrability nevertheless ensures that the solutions derived are structurally robust and belong to a well-defined analytical framework. The parameter $\mu$ in Eq. (1) plays a critical physical role, governing the strength of two competing effects: the fifth-order dispersion term $\mu u_{xxxxx}$, which tends to spread wave packets and introduce phase modulation, and the mixed dispersive-advective term $\mu u_x$, which contributes to wave propagation speed. The balance between these $\mu$-dependent terms and the nonlinear self-steepening terms $(u u_{xxx} + u u_x + 3 u_x u_{xx})$ determines the shape, amplitude, and stability of traveling wave solutions. Large values of $|\mu |$ promote stronger dispersion, leading to broader, more oscillatory wave profiles, while small $|\mu |$ allows nonlinearity to dominate, favoring narrow, steep-fronted solitary structures. This interplay is central to understanding the diversity of solution types derived in Section Derivation of exact traveling-wave solutions. The present research applies the modified extended mapping method (MEMM)^26^ to derive a broader array of exact traveling wave solutions for the $(1+1)$-dimensional Wazwaz fifth-order equation. This approach utilizes a series expansion with a mapping function to systematically obtain solitons, periodic waves, singular structures, and other forms, thereby complementing and expanding upon Wazwaz’s results. The MEMM’s adaptability effectively handles the equation’s high-order derivatives and nonlinearities, providing new analytical perspectives on wave propagation. The novelty of the present work relative to^25^ and to the broader literature on fifth-order nonlinear evolution equations may be summarized as follows. First, this study represents the first systematic application of the MEMM to the Wazwaz fifth-order equation, yielding solution classes, including Weierstrass elliptic, Jacobi elliptic, combined bright–dark solitons, rational, and exponential solutions, that were not derived in^25^ and which capture more complex periodic, quasi-periodic, and transient wave behaviors. Second, the unified auxiliary-equation framework of the MEMM encompasses all previously known solution types (tanh-based kinks, ${{\,\textrm{sech}\,}}^2$ bright solitons, tan-based periodic waves) as special parametric limits while simultaneously generating new families through systematic variation of the six coefficients $(c_0,\ldots ,c_5)$. Third, the linear stability analysis establishes the dispersion relation for perturbed states and identifies parameter regimes governing wave propagation characteristics, thereby complementing the exact solution catalogue with dynamical insights. Collectively, these contributions provide a comprehensive analytical foundation for understanding the full spectrum of traveling wave solutions admitted by this recently introduced Painlevé-integrable model.

The present paper is structured across five principal sections. Section Introduction introduces the study, delineating the background, motivation, and primary objectives. Section MEMM is devoted to a comprehensive description of the modified extended mapping method (MEMM). In Section Derivation of exact traveling-wave solutions, we systematically derive and classify the exact traveling wave solutions obtained for the (1+1)-dimensional Wazwaz fifth-order equation. Section An examination of instability in the suggested model examines the instability of the model. Section Visual analysis of selected solutions offers an extensive graphical analysis through three-dimensional, two-dimensional, and contour plots that illustrate the physical characteristics and spatiotemporal behavior of selected solutions. Finally, Section Conclusion summarizes the main achievements, discusses their significance, and outlines potential avenues for future investigation.

## MEMM

The MEMM provides a powerful and systematic procedure for deriving exact traveling-wave solutions of NPDEs. The main steps of the method are described as follows^26^:

**Phase I:** Consider a general NPDE expressed as

2 $$\begin{aligned} F(u,u_t,u_x,u_{xx},u_{xxx},u_{xxxx},u_{xxxxx},\dots )=0, \end{aligned}$$

where $u=u(x,t)$ is the unknown function and $F$ is a polynomial in $u$ and its partial derivatives.

**Phase II:** Introduce the traveling-wave transformation

3 $$\begin{aligned} u(x,t)=\Upsilon (\xi ),\quad \xi =x-at,\quad a\in \mathbb {R}\setminus \{0\}, \end{aligned}$$

in which $a$ denotes the wave speed. Substituting (3) into (2) reduces the equation to an ordinary differential equation (ODE)

4 $$\begin{aligned} G(\Upsilon ,\Upsilon ',\Upsilon '',\Upsilon ''',\dots )=0. \end{aligned}$$

**Phase III:** Assume a solution of the form

5 $$\begin{aligned} \Upsilon (\xi )=\sum _{i=0}^{N}p_i\,\mathcal {W}^i(\xi ) +\sum _{i=1}^{N}q_i\,\mathcal {W}^{-i}(\xi ) +\sum _{i=2}^{N}r_i\,\mathcal {W}^{i-2}(\xi )\,\mathcal {W}'(\xi ) +\sum _{i=1}^{N}s_i\,\frac{\mathcal {W}'(\xi )}{\mathcal {W}^i(\xi )}, \end{aligned}$$

where $p_i,q_i,r_i,s_i$ are constants to be determined, and the auxiliary function $\mathcal {W}(\xi )$ satisfies

6 $$\begin{aligned} \mathcal {W}'(\xi )=\sqrt{c_0+c_1\mathcal {W}+c_2\mathcal {W}^2+c_3\mathcal {W}^3+c_4\mathcal {W}^4+c_5\mathcal {W}^6}, \end{aligned}$$

with real coefficients $c_j$ ($j=0,1,\dots ,5$).

**Phase IV:** Determine the positive integer $N$ by balancing the highest-order derivative term with the leading nonlinear term in the ODE (4).

**Phase V:** Insert the ansatz (5) together with the relation (6) into the ODE (4). This yields a polynomial in powers of $\mathcal {W}$ (and terms involving $\mathcal {W}'$). Setting the coefficients of all independent terms to zero produces a system of algebraic equations for the unknowns $p_i,q_i,r_i,s_i,a$ and the parameters $c_j$. Solving this system using symbolic software such as Maple, Mathematica, or MATLAB delivers explicit parameter values, from which a broad spectrum of exact solutions, including hyperbolic, trigonometric, elliptic, exponential, and rational functions, are obtained for the original NPDE (2). The MEMM is structurally distinct from the standard $G'/G$-expansion^27^ and its extended Riccati-type variants in three concrete respects that carry direct mathematical consequences. First, the auxiliary equation (6) is a first-order ODE with a polynomial radicand of degree up to six. When $c_5=0$ and the radicand is quartic, its general solution is a Jacobi elliptic function; when $c_2=c_4=c_5=0$ and the radicand is cubic, it reduces to the Weierstrass $\wp$-function. Neither the $G'/G$ method nor any Riccati-based extension can generate these doubly periodic solution classes because their auxiliary equations do not reduce to the Jacobi or Weierstrass normal forms. Second, the ansatz (5) contains cross terms of the form $\mathcal {W}^{i-2}\mathcal {W}'$ and $\mathcal {W}'/\mathcal {W}^i$, which are absent from all $G'/G$-type ansätze; these mixed derivative–power terms are indispensable for generating the combined bright–dark soliton structures reported in Section "Derivation of exact traveling-wave solutions" (e.g., solutions (38)–(40)). Third, the unified auxiliary-equation framework requires solving only one algebraic system to cover all solution families simultaneously, whereas $G'/G$ extensions typically require separate treatments for hyperbolic, trigonometric, and rational branches. This unified framework is computationally more efficient, particularly for higher-order equations such as Eq. (1).

### Scope and limitations of the MEMM

The MEMM operates exclusively within the traveling-wave class $u(x,t)=\Upsilon (x-\eta t)$; solutions that are not of traveling-wave type lie outside its scope. Within this class, valid solutions arise only for parameter combinations $(c_i,\eta ,\mu )$ that satisfy the full overdetermined algebraic system of Phase V simultaneously; arbitrary coefficient choices do not generally yield solutions. The method is not proven to be complete: solutions expressible only through logarithmic, transcendental, or non-algebraic combinations of $\mathcal {W}(\xi )$ cannot be recovered by the present ansatz. Concerning stability, the MEMM establishes the existence and functional form of exact solutions but provides no information about their orbital or Lyapunov stability; a rigorous stability analysis of the soliton and cnoidal solutions derived here would require variational or spectral methods and is identified as an important direction for future work. Finally, while the method extends naturally to equations with polynomial nonlinearities and integer-order derivatives, equations involving non-local interactions, non-polynomial nonlinearities, or fractional-order operators require problem-specific modifications of both the auxiliary equation and the ansatz before the MEMM framework can be applied.

## Derivation of exact traveling-wave solutions

In this section, we systematically apply the MEMM to construct exact propagating-wave solutions for the (1+1)-dimensional Wazwaz fifth-order equation (1). We begin by employing the standard traveling-wave reduction

7 $$\begin{aligned} u(x,t)=\Upsilon (\xi ),\quad \xi =x-\eta t, \end{aligned}$$

where the constant $\eta$ represents the unknown wave velocity.

Inserting (7) into (1) and carrying out the required differentiations produces the fifth-order ordinary differential equation

8 $$\begin{aligned} -\Upsilon '(\eta -\mu +\Upsilon +3\Upsilon '')-(\eta +\Upsilon )\Upsilon '''-\mu \Upsilon '''''=0. \end{aligned}$$

Integrating (8) once with respect to $\xi$ and setting the integration constant to zero (justified by the localized nature of the expected solutions) yields the reduced fourth-order equation

9 $$\begin{aligned} (-\eta +\mu )\Upsilon -\frac{1}{2}\Upsilon ^2-(\Upsilon ')^2-\eta \Upsilon ''-\Upsilon \Upsilon ''-\mu \Upsilon ''''=0. \end{aligned}$$

Following the MEMM protocol, we express the solution of (9) in the general form (5). Balancing the highest-order derivative term $(\Upsilon ')^2$ with the dominant nonlinear term $\Upsilon ''''$ gives $N=2$^28^. Consequently, the ansatz truncates to

10 $$\begin{aligned} \begin{aligned} \Upsilon (\xi )=\;p_0+p_1\mathcal {W}(\xi )+p_2\mathcal {W}^2(\xi )+\frac{q_1}{\mathcal {W}(\xi )}+\frac{q_2}{\mathcal {W}^2(\xi )} +r_2\mathcal {W}'(\xi )+s_1\frac{\mathcal {W}'(\xi )}{\mathcal {W}(\xi )}+s_2\frac{\mathcal {W}'(\xi )}{\mathcal {W}^2(\xi )}, \end{aligned} \end{aligned}$$

where $p_0,p_1,p_2,q_1,q_2,r_2,s_1,s_2$ are undetermined real coefficients.

Substituting (10) together with the auxiliary relation (6) into (9) transforms the equation into a polynomial in $\mathcal {W}$ and $\mathcal {W}'$. Collecting terms according to powers $\mathcal {W}^i$ ($i\in [-6,10]$) and $\mathcal {W}^j\mathcal {W}'$ ($j\in [-6,14]$) produces a total of 38 independent coefficients. Setting each coefficient to zero generates an over-determined algebraic system in the unknown parameters $p_k,q_k,r_2,s_k,\eta ,\mu$ and the auxiliary constants $c_j$ ($j=0,\dots ,5$).

Solving this system symbolically yields several consistent parameter families, each corresponding to distinct classes of exact solutions for the original PDE (1). These solutions, presented in the subsequent case studies, encompass bright and dark solitons, singular and regular periodic waves, rational functions, exponential forms, combined soliton structures, as well as Weierstrass and Jacobi elliptic solutions. All derived solutions have been verified by direct substitution into Eq. (1) using *Wolfram Mathematica*, confirming their exactness. Among the solutions derived below, several exhibit singularities (poles) at finite spatial or temporal points. These *singular solitons* and *singular periodic waves* are characterized by solution profiles that diverge to infinity at isolated values of the argument $\xi = x - \eta t$. While such solutions are not physically realizable in smooth continuous media, they arise naturally within the MEMM framework for specific parameter combinations and possess both mathematical and physical significance. Mathematically, they represent limiting cases of the auxiliary equation (6) where the polynomial under the square root admits roots, leading to logarithmic or inverse-trigonometric integrations that produce poles. Physically, singular solutions can model wave-breaking events, shock-front formation, current sheets in plasma physics, or boundary-layer structures in fluid dynamics, phenomena where the continuum approximation underlying Eq. (1) breaks down and the solution exhibits rapid, localized gradients. Their inclusion in the solution catalogue ensures completeness and aids in understanding the parametric boundaries between regular and singular behavior.

**Case Study 1:** $$c_0 = c_1 = c_3 = c_5 = 0$$

In this family, the constant ($c_0$), linear ($c_1$), cubic ($c_3$), and sextic ($c_5$) terms in auxiliary equation (6) are set to zero, so that the auxiliary equation reduces to $\mathcal {W}' = \sqrt{c_2\mathcal {W}^2 + c_4\mathcal {W}^4}$. This biparametric reduction is integrable in closed form: when $c_2> 0$ and $c_4 < 0$, the solution involves hyperbolic functions and yields localized (soliton-type) wave profiles, while $c_2 < 0$ and $c_4> 0$ leads to trigonometric functions and periodic or singular periodic structures. Solving the over-determined algebraic system generated by the MEMM yields three consistent parameter sets, each producing a qualitatively distinct class of exact solutions.

$${\textbf {[1.1]}}$$ $$p_0 = -\frac{8 \eta c_2}{1 + 4 c_2}, \quad p_1 = 0, \quad p_2 = -\frac{12 \eta c_4}{1 + 4 c_2}, \quad q_1 = q_2 = r_2 = s_1 = s_2 = 0, \quad \mu = \frac{\eta }{1 + 4 c_2}.$$

$${\textbf {[1.2]}}$$ $$p_0 = p_1 = 0, \quad p_2 = \frac{6 \eta c_4}{-1 + c_2}, \quad q_1 = q_2 = 0, \quad r_2 = -\frac{6 \eta \sqrt{c_4}}{\sqrt{1 - 2 c_2 + c_2^2}}, \quad s_1 = s_2 = 0, \quad \mu = -\frac{\eta }{-1 + c_2}.$$

$${\textbf {[1.3]}}$$ $$p_0 = -\frac{2 \eta c_2}{1 + c_2}, \quad p_1 = 0, \quad p_2 = -\frac{6 \eta c_4}{1 + c_2}, \quad q_1 = q_2 = 0, \quad r_2 = -\frac{6 \eta \sqrt{c_4}}{\sqrt{1 + 2 c_2 + c_2^2}}, \quad s_1 = s_2 = 0, \quad \mu = \frac{\eta }{1 + c_2}.$$

From Subcase [1.1] the following explicit solutions are obtained:

${\textbf {[1.1.1]}}$ If $c_2> 0$, and $c_4 < 0$, then

11 $$\begin{aligned} u_{1.1.1}(x, t) = \frac{4 \, \eta \, c_2 \left( -2 + 3 {{\,\textrm{sech}\,}}^2\left[ \sqrt{c_2} (x - \eta t)\right] \right) }{1 + 4 c_2}, \end{aligned}$$

describes a bright soliton.

${\textbf {[1.1.2]}}$ If $c_2 < 0$, and $c_4> 0$, then

12 $$\begin{aligned} u_{1.1.2}(x, t) = \frac{4 \, \eta \, c_2 \left( -2 + 3 \sec ^2\left[ \sqrt{-c_2} (x - \eta t)\right] \right) }{1 + 4 c_2}, \end{aligned}$$

13 $$\begin{aligned} u_{1.1.3}(x, t) = \frac{4 \, \eta \, c_2 \left( -2 + 3 \csc ^2\left[ \sqrt{-c_2} (x - \eta t)\right] \right) }{1 + 4 c_2}, \end{aligned}$$

describe singular periodic.

From Subcase [1.2] the following explicit solutions are obtained:

${\textbf {[1.2.1]}}$ If $c_2 < 0$, and $c_4> 0$, then

14 $$\begin{aligned} u_{1.2.1}(x, t) = -\frac{6 \, \eta \, c_2 \sec ^2\left[ \sqrt{-c_2} (x - \eta t)\right] \left( 1 + \sin \left[ \sqrt{-c_2} (x - \eta t)\right] \right) }{ c_2 - 1}, \end{aligned}$$

describes a singular periodic.

${\textbf {[1.2.2]}}$ If $c_2 < 0$, and $c_4> 0$, then

15 $$\begin{aligned} u_{1.2.2}(x, t) = -\frac{3 \, \eta \, c_2 \sec ^2\left[ \frac{1}{2} \sqrt{-c_2} (x - \eta t)\right] }{ c_2 - 1}, \end{aligned}$$

describes a singular periodic.

From Subcase [1.3] the following explicit solutions are obtained:

${\textbf {[1.3.1]}}$ If $c_2 < 0$, and $c_4> 0$, then

16 $$\begin{aligned} u_{1.3.1}(x, t) = -\frac{2 \, \eta \, c_2 \left( 2 + \sin \left[ \sqrt{-c_2} (x - \eta t)\right] \right) }{\left( -1 + \sin \left[ \sqrt{-c_2} (x - \eta t)\right] \right) \left( 1 + c_2\right) }, \end{aligned}$$

yields a periodic solution.

${\textbf {[1.3.2]}}$ If $c_2 < 0$, and $c_4> 0$, then

17 $$\begin{aligned} u_{1.3.2}(x, t) = -\frac{ \, \eta \, c_2 \left( -2 + \cos \left[ \sqrt{-c_2} (x - \eta t)\right] \right) \sec ^2\left[ \frac{1}{2} \sqrt{-c_2} (x - \eta t)\right] }{1 + c_2}, \end{aligned}$$

describes a singular periodic.

**Case Study 2:** $$c_1 = c_3 = c_5 = 0$$

Here only the linear ($c_1$), cubic ($c_3$), and sextic ($c_5$) coefficients are suppressed, so the auxiliary equation takes the form $\mathcal {W}' = \sqrt{c_0 + c_2\mathcal {W}^2 + c_4\mathcal {W}^4}$. This is a standard elliptic-type auxiliary equation whose solutions are expressible in terms of Jacobi elliptic functions in the general case and degenerate into hyperbolic or trigonometric forms when the discriminant $c_2^2 - 4c_0 c_4$ takes special values. The special constraint $c_0 = c_2^2/(4c_4)$ corresponds to a perfect-square factorization of the radicand, producing $\text {csch}^2$- and $\text {csc}^2$-type solutions. Five consistent parameter families emerge from the algebraic system.

$${\textbf {[2.1]}}$$ $$p_0 = -6 \mu c_2, \quad p_1 = p_2 = q_1 = 0, \quad q_2 = -\frac{3 \mu c_2^2}{c_4}, \quad r_2 = s_1 = s_2 = 0, \quad \eta = \mu + 2 \mu c_2,$$

$${\textbf {[2.2]}}$$ $$p_0 = -12 \mu c_2, \quad p_1 = 0, \quad p_2 = -12 \mu c_4, \quad q_1 = 0, \quad q_2 = -\frac{3 \mu c_2^2}{c_4}, \quad r_2 = s_1 = s_2 = 0, \quad \eta = \mu + 8 \mu c_2.$$

$${\textbf {[2.3]}}$$ $$p_0 = -3 \mu c_2, \quad p_1 = 0, \quad p_2 = -6 \mu c_4, \quad q_1 = q_2 = 0, \quad r_2 = -6 \mu \sqrt{c_4}, \quad s_1 = s_2 = 0, \quad \eta = \mu + 2 \mu c_2.$$

$${\textbf {[2.4]}}$$ $$p_0 = -3 \mu c_2, \quad p_1 = p_2 = q_1 = 0, \quad q_2 = -\frac{3 \mu c_2^2}{2 c_4}, \quad r_2 = s_1 = 0, \quad s_2 = -\frac{3 \mu c_2}{\sqrt{c_4}}, \quad \eta = \mu + 2 \mu c_2.$$

$${\textbf {[2.5]}}$$ $$p_0 = -6 \mu c_2, \quad p_1 = 0, \quad p_2 = -6 \mu c_4, \quad q_1 = 0, \quad q_2 = -\frac{3 \mu c_2^2}{2 c_4}, \quad r_2 = -6 \mu \sqrt{c_4}, \quad s_1 = 0, \quad s_2 = -\frac{3 \mu c_2}{\sqrt{c_4}}, \quad \eta = \mu + 8 \mu c_2.$$

From Subcase [2.1] the following explicit solutions are obtained:

${\textbf {[2.1.1]}}$ If $c_2 < 0$, $c_4> 0$, and $c_0 = \frac{c_2^2}{4 c_4}$, then

18 $$\begin{aligned} u_{2.1.1}(x, t) = 6 \, \mu \, c_2 {{\,\textrm{csch}\,}}^2\left[ \frac{\sqrt{-c_2}}{\sqrt{2}} (x - \eta t)\right] , \end{aligned}$$

describes a singular soliton.

${\textbf {[2.1.2]}}$ If $c_2> 0$, $c_4> 0$, and $c_0 = \frac{c_2^2}{4 c_4}$, then

19 $$\begin{aligned} u_{2.1.2}(x, t) = -6 \, \mu \, c_2 \csc ^2\left[ \frac{\sqrt{c_2}}{\sqrt{2}} (x - \eta t)\right] , \end{aligned}$$

describes a singular periodic.

From Subcase [2.2] the following explicit solutions are obtained:

${\textbf {[2.2.1]}}$ If $c_2 < 0$, $c_4> 0$, and $c_0 = \frac{c_2^2}{4 c_4}$, then

20 $$\begin{aligned} u_{2.2.1}(x, t) = 24 \, \mu \, c_2 {{\,\textrm{csch}\,}}^2\left[ \sqrt{2} \sqrt{-c_2} (x - \eta t)\right] , \end{aligned}$$

describes a singular soliton.

${\textbf {[2.2.2]}}$ If $c_2> 0$, $c_4> 0$, and $c_0 = \frac{c_2^2}{4 c_4}$, then

21 $$\begin{aligned} u_{2.2.2}(x, t) = -24 \, \mu \, c_2 \csc ^2\left[ \sqrt{2} \sqrt{c_2} (x - \eta t)\right] , \end{aligned}$$

describes a singular periodic.

From Subcase [2.3] the following explicit solutions are obtained:

${\textbf {[2.3.1]}}$ If $c_2> 0$, $c_4> 0$, and $c_0 = \frac{c_2^2}{4 c_4}$, then

22 $$\begin{aligned} u_{2.3.1}(x, t) = -6 \, \mu \, c_2 \sec ^2\left[ \frac{\sqrt{c_2}}{\sqrt{2}} (x - \eta t)\right] , \end{aligned}$$

describes a singular periodic.

From Subcase [2.4] the following explicit solutions are obtained:

${\textbf {[2.4.1]}}$ If $c_2> 0$, $c_4> 0$, and $c_0 = \frac{c_2^2}{4 c_4}$, then

23 $$\begin{aligned} u_{2.4.1}(x, t) = -6 \, \mu \, c_2 \csc ^2\left[ \frac{\sqrt{c_2}}{\sqrt{2}} (x - \eta t)\right] , \end{aligned}$$

describes a singular periodic.

From Subcase [2.5] the following explicit solution are obtained:

${\textbf {[2.5.1]}}$ If $c_2> 0$, $c_4> 0$, and $c_0 = \frac{c_2^2}{4 c_4}$, then

24 $$\begin{aligned} u_{2.5.1}(x, t) = -24 \, \mu \, c_2 \csc ^2\left[ \sqrt{2} \sqrt{c_2} (x - \eta t)\right] , \end{aligned}$$

describes a singular periodic.

**Case Study 3:** $$c_3 = c_4 = c_5 = 0$$

In this case, the cubic ($c_3$), quartic ($c_4$), and sextic ($c_5$) terms are removed, reducing the auxiliary equation to $\mathcal {W}' = \sqrt{c_0 + c_1\mathcal {W} + c_2\mathcal {W}^2}$, a quadratic radicand that integrates to exponential or trigonometric functions depending on the sign of the discriminant $c_1^2 - 4c_0 c_2$. The choice $c_0 = c_1^2/(4c_2)$ (perfect-square case) leads to exponential solutions (Subcases [3.5] and [3.6]), while the generic case with $c_1 = 0$ recovers soliton and singular periodic profiles through $\text {sech}^2$ and $\text {csc}^2$ functions. Six parameter families are obtained.

$${\textbf {[3.1]}}$$ $$p_0 = p_1 = p_2 = q_1 = 0, \quad q_2 = \frac{12 \eta c_0}{-1 + 4 c_2}, \quad r_2 = s_1 = s_2 = 0, \quad \mu = -\frac{\eta }{-1 + 4 c_2}, \quad c_1 = 0.$$

$${\textbf {[3.2]}}$$ $$p_0 = -\frac{8 \eta c_2}{1 + 4 c_2}, \quad p_1 = p_2 = q_1 = 0, \quad q_2 = -\frac{12 \eta c_0}{1 + 4 c_2}, \quad r_2 = s_1 = s_2 = 0, \quad \mu = \frac{\eta }{1 + 4 c_2}, \quad c_1 = 0.$$

$${\textbf {[3.3]}}$$ $$p_0 = p_1 = p_2 = q_1 = 0, \quad q_2 = \frac{6 \eta c_0}{-1 + c_2}, \quad r_2 = s_1 = 0, \quad s_2 = \frac{6 \eta \sqrt{c_0}}{\sqrt{1 - 2 c_2 + c_2^2}}, \quad \mu = -\frac{\eta }{-1 + c_2}, \quad c_1 = 0.$$

$${\textbf {[3.4]}}$$ $$p_0 = -\frac{2 \eta c_2}{1 + c_2}, \quad p_1 = p_2 = q_1 = 0, \quad q_2 = -\frac{6 \eta c_0}{1 + c_2}, \quad r_2 = s_1 = 0, \quad s_2 = \frac{6 \eta \sqrt{c_0}}{\sqrt{1 + 2 c_2 + c_2^2}}, \quad \mu = \frac{\eta }{1 + c_2}, \quad c_1 = 0.$$

$${\textbf {[3.5]}}$$ $$p_0 = p_1 = p_2 = 0, \quad q_1 = \sqrt{c_2}, \quad q_2 = \frac{c_1}{2 \sqrt{c_2}}, \quad r_2 = s_1 = 0, \quad s_2 = 1, \quad \mu = -\frac{\sqrt{c_2}}{3 c_1}, \quad \eta = \frac{-\sqrt{c_2} + c_2^{3/2}}{3 c_1}.$$

$${\textbf {[3.6]}}$$ $$p_0 = \frac{2 c_2^{3/2}}{3 c_1}, \quad p_1 = p_2 = 0, \quad q_1 = \sqrt{c_2}, \quad q_2 = \frac{c_1}{2 \sqrt{c_2}}, \quad r_2 = s_1 = 0, \quad s_2 = 1, \quad \mu = -\frac{\sqrt{c_2}}{3 c_1}, \quad \eta = \frac{-\sqrt{c_2} - c_2^{3/2}}{3 c_1}.$$

From Subcase [3.1] the following explicit solutions are obtained:

${\textbf {[3.1.1]}}$ If $c_2> 0$, $c_0> 0$, and $c_1 = 0$, then

25 $$\begin{aligned} u_{3.1.1}(x, t) = \frac{12 \, \eta \, c_2 {{\,\textrm{csch}\,}}^2\left[ \sqrt{c_2} (x - \eta t)\right] }{-1 + 4 c_2}, \end{aligned}$$

describes a singular soliton.

${\textbf {[3.1.2]}}$ If $c_2 < 0$, $c_0> 0$, and $c_1 = 0$, then

26 $$\begin{aligned} u_{3.1.2}(x, t) = \frac{12 \, \eta \, c_2 \csc ^2\left[ \sqrt{-c_2} (x - \eta t)\right] }{1 - 4 c_2}, \end{aligned}$$

describes a singular periodic.

From Subcase [3.2] the following explicit solutions are obtained:

${\textbf {[3.2.1]}}$ If $c_2> 0$, $c_0> 0$, and $c_1 = 0$, then

27 $$\begin{aligned} u_{3.2.1}(x, t) = -\frac{4 \, \eta \, c_2 \left( 2 + 3 {{\,\textrm{csch}\,}}^2\left[ \sqrt{c_2} (x - \eta t)\right] \right) }{1 + 4 c_2}, \end{aligned}$$

describes a singular soliton.

${\textbf {[3.2.2]}}$ If $c_2 < 0$, $c_0> 0$, and $c_1 = 0$, then

28 $$\begin{aligned} u_{3.2.2}(x, t) = \frac{4 \, \eta \, c_2 \left( -2 + 3 \csc ^2\left[ \sqrt{-c_2} (x - \eta t)\right] \right) }{1 + 4 c_2}, \end{aligned}$$

describes a singular periodic.

From Subcase [3.3] the following explicit solutions are obtained:

${\textbf {[3.3.1]}}$ If $c_2> 0$, $c_0> 0$, and $c_1 = 0$, then

29 $$\begin{aligned} u_{3.3.1}(x, t) = -\frac{3 \, \eta \, c_2 {{\,\textrm{sech}\,}}^2\left[ \frac{\sqrt{c_2}}{2} (x - \eta t)\right] }{-1 + c_2}, \end{aligned}$$

describes a bright soliton.

${\textbf {[3.3.2]}}$ If $c_2 < 0$, $c_0> 0$, and $c_1 = 0$, then

30 $$\begin{aligned} u_{3.3.2}(x, t) = -\frac{3 \, \eta \, c_2 \sec ^2\left[ \frac{\sqrt{-c_2}}{2} (x - \eta t)\right] }{-1 + c_2}, \end{aligned}$$

describes a singular periodic.

From Subcase [3.4] the following explicit solutions are obtained:

${\textbf {[3.4.1]}}$ If $c_2> 0$, $c_0> 0$, and $c_1 = 0$, then

31 $$\begin{aligned} u_{3.4.1}(x, t) = -\frac{\, \eta \, c_2 \left( -2 + \cosh \left[ \sqrt{c_2} (x - \eta t)\right] \right) {{\,\textrm{sech}\,}}^2\left[ \frac{\sqrt{c_2}}{2} (x - \eta t)\right] }{1 + c_2}, \end{aligned}$$

yields a hyperbolic.

${\textbf {[3.4.2]}}$ If $c_2 < 0$, $c_0> 0$, and $c_1 = 0$, then

32 $$\begin{aligned} u_{3.4.2}(x, t) = -\frac{\, \eta \, c_2 \left( -2 + \cos \left[ \sqrt{-c_2} (x - \eta t)\right] \right) \sec ^2\left[ \frac{\sqrt{-c_2}}{2} (x - \eta t)\right] }{1 + c_2}, \end{aligned}$$

describes a singular periodic.

From Subcase [3.5] the following explicit solution are obtained:

${\textbf {[3.5.1]}}$ If $c_2> 0$ and $c_0 = \frac{c_1^2}{4 c_2}$, then

33 $$\begin{aligned} u_{3.5.1}(x, t) = \frac{8 \, c_2^{5/2} e^{\sqrt{c_2} (x - \eta t)} }{\left( c_1 - 2 \, c_2 \, e^{\sqrt{c_2} (x - \eta t)} \right) ^2}, \end{aligned}$$

yields an exponential.

From Subcase [3.6] the following explicit solution are obtained:

${\textbf {[3.6.1]}}$ If $c_2> 0$ and $c_0 = \frac{c_1^2}{4 c_2}$, then

34 $$\begin{aligned} u_{3.6.1}(x, t) = \frac{2 \, c_2^{3/2} \left( c_1^2 + 8 \, c_1 \, c_2 \, e^{\sqrt{c_2} (x - \eta t)} + 4 \, c_2^2 \, e^{2 \sqrt{c_2} (x - \eta t)} \right) }{3 c_1 \left( c_1 - 2 \, c_2 \, e^{\sqrt{c_2} (x - \eta t)} \right) ^2}, \end{aligned}$$

yields an exponential.

**Case Study 4:** $$c_0 = c_1 = c_2 = c_5 = 0$$

Setting the four lowest-order coefficients to zero leaves the auxiliary equation as $\mathcal {W}' = \sqrt{c_3\mathcal {W}^3 + c_4\mathcal {W}^4}$, a purely cubic-quartic radicand. Upon integration and substitution into the ansatz, this choice generates a rational traveling-wave solution whose amplitude decays algebraically rather than exponentially, in contrast to soliton profiles. Only one consistent parameter set is obtained in this case.

$${\textbf {[4.1]}}$$ $$p_0 = 0, \quad p_1 = -3 \mu c_3, \quad p_2 = -6 \mu c_4, \quad q_1 = q_2 = s_1 = s_2 = 0, \quad r_2 = -6 \mu \sqrt{c_4}, \quad \eta = \mu .$$

From Subcase [4.1] the following explicit solution are obtained:

$${\textbf {[4.1.1]}}$$ If $$c_4 \ne 0$$, then

35 $$\begin{aligned} u_{4.1.1}(x, t) = -\frac{12 \mu c_3^2}{\left( (x - \eta t) c_3 + 2 \sqrt{c_4} \right) ^2}, \end{aligned}$$

yields a rational.

**Case Study 5:** $$c_0 = c_1 = c_5 = 0$$

Here the constant ($c_0$), linear ($c_1$), and sextic ($c_5$) terms vanish, so $\mathcal {W}' = \sqrt{c_2\mathcal {W}^2 + c_3\mathcal {W}^3 + c_4\mathcal {W}^4}$. The cubic term $c_3\mathcal {W}^3$ breaks the even symmetry of the radicand present in Case Studies 1 and 2, enabling the derivation of dark solitons and combined bright–dark soliton structures that are not accessible when $c_3 = 0$. The parameter constraint $c_2 = c_3^2/(4c_4)$ corresponds to a degenerate factorization yielding tanh- and coth-type solutions. Five parameter families arise, producing the richest variety of solution types in the present study.

$${\textbf {[5.1]}}$$ $$p_0 = -\frac{\mu c_3^2}{2 c_4}, \quad p_1 = -6 \mu c_3, \quad p_2 = -12 \mu c_4, \quad q_1 = q_2 = r_2 = s_1 = s_2 = 0, \quad \eta = \frac{\mu \left( c_3^2 + 4 c_4 \right) }{4 c_4}.$$

$${\textbf {[5.2]}}$$ $$p_0 = 0, \quad p_1 = -3 \mu c_3, \quad p_2 = -6 \mu c_4, \quad q_1 = q_2 = 0, \quad r_2 = -6 \mu \sqrt{c_4}, \quad s_1 = s_2 = 0, \quad \eta = \frac{-\mu c_3^2 + 4 \mu c_4}{4 c_4}.$$

$${\textbf {[5.3]}}$$ $$p_0 = -\frac{\mu c_3^2}{2 c_4}, \quad p_1 = -3 \mu c_3, \quad p_2 = -6 \mu c_4, \quad q_1 = q_2 = 0, \quad r_2 = -6 \mu \sqrt{c_4}, \quad s_1 = s_2 = 0, \quad \eta = \frac{\mu \left( c_3^2 + 4 c_4 \right) }{4 c_4}.$$

$${\textbf {[5.4]}}$$ $$p_0 = 0, \quad p_1 = -3 \mu c_3, \quad p_2 = -6 \mu c_4, \quad q_1 = q_2 = 0, \quad r_2 = 6 \mu \sqrt{c_4}, \quad s_1 = s_2 = 0, \quad \eta = \mu - \mu c_2.$$

$${\textbf {[5.5]}}$$ $$p_0 = -2 \mu c_2, \quad p_1 = -3 \mu c_3, \quad p_2 = -6 \mu c_4, \quad q_1 = q_2 = 0, \quad r_2 = 6 \mu \sqrt{c_4}, \quad s_1 = s_2 = 0, \quad \eta = \mu (1 + c_2).$$

From Subcase [5.1] the following explicit solutions are obtained:

${\textbf {[5.1.1]}}$ If $c_2> 0$ and $c_2 = \frac{c_3^2}{4 c_4}$, then

36 $$\begin{aligned} u_{5.1.1}(x, t) = -\frac{\mu c_3^2}{2 c_4} + 6 \mu c_2 \left( 1 + \tanh \left[ \frac{\sqrt{c_2}}{2} (x - \eta t) \right] \right) - \frac{12 \mu c_2^2 c_4 \left( 1 + \tanh \left[ \frac{\sqrt{c_2}}{2} (x - \eta t) \right] \right) ^2}{c_3^2}, \end{aligned}$$

describes a dark soliton.

${\textbf {[5.1.2]}}$ If $c_2> 0$ and $c_2 = \frac{c_3^2}{4 c_4}$, then

37 $$\begin{aligned} u_{5.1.2}(x, t) = 6 \mu \left( 1 + \coth \left[ \frac{\sqrt{c_2}}{2} (x - \eta t) \right] \right) c_2 - \frac{\mu c_3^2}{2 c_4} - \frac{12 \mu c_2^2 c_4 \left( 1 + \coth \left[ \frac{\sqrt{c_2}}{2} (x - \eta t) \right] \right) ^2 }{c_3^2}, \end{aligned}$$

describes a singular soliton.

From Subcase [5.2] the following explicit solutions are obtained:

${\textbf {[5.2.1]}}$ If $c_2> 0$ and $c_2 = \frac{c_3^2}{4 c_4}$, then

38 $$\begin{aligned} \begin{aligned} u_{5.2.1}(x, t) = \frac{3 \mu c_2}{c_3^2} \Big (c_3 \sqrt{c_2 c_4} \, {{\,\textrm{sech}\,}}^2\left[ \frac{\sqrt{c_2}}{2} (x - \eta t) \right] + c_3^2 \left( 1 + \tanh \left[ \frac{\sqrt{c_2}}{2} (x - \eta t) \right] \right) \\ - 2 c_2 c_4 \left( 1 + \tanh \left[ \frac{\sqrt{c_2}}{2} (x - \eta t) \right] \right) ^2 \Big ), \end{aligned} \end{aligned}$$

describes a combo bright-dark soliton.

${\textbf {[5.2.2]}}$ If $c_2> 0$ and $c_2 = \frac{c_3^2}{4 c_4}$, then

39 $$\begin{aligned} \begin{aligned} u_{5.2.2}(x, t) = -\frac{3 \mu c_2}{c_3^2} \Big ( -\left( 1 + \coth \left[ \frac{\sqrt{c_2}}{2} (x - \eta t) \right] \right) c_3^2 + 2 c_2 c_4 \left( 1 + \coth \left[ \frac{\sqrt{c_2}}{2} (x - \eta t) \right] \right) ^2 \\ + c_3 \sqrt{c_2 c_4} \, {{\,\textrm{csch}\,}}^2\left[ \frac{\sqrt{c_2}}{2} (x - \eta t) \right] \Big ), \end{aligned} \end{aligned}$$

describes a singular soliton.

From Subcase [5.3] the following explicit solutions are obtained:

${\textbf {[5.3.1]}}$ If $c_2> 0$ and $c_2 = \frac{c_3^2}{4 c_4}$, then

40 $$\begin{aligned} \begin{aligned} u_{5.3.1}(x, t) = -\frac{\mu c_3^2}{2 c_4} - \frac{6 \mu c_2^2 c_4 \left( 1 + \tanh \left[ \frac{\sqrt{c_2}}{2} (x - \eta t) \right] \right) ^2}{c_3^2} \\ + 3 \mu c_2 \left( 1 + \frac{ \sqrt{c_2 c_4} \, {{\,\textrm{sech}\,}}^2\left[ \frac{\sqrt{c_2}}{2} (x - \eta t) \right] }{c_3} + \tanh \left[ \frac{\sqrt{c_2}}{2} (x - \eta t) \right] \right) , \end{aligned} \end{aligned}$$

describes a combo bright-dark soliton.

${\textbf {[5.3.2]}}$ If $c_2> 0$ and $c_2 = \frac{c_3^2}{4 c_4}$, then

41 $$\begin{aligned} \begin{aligned} u_{5.3.2}(x, t) = -\frac{\mu c_3^2}{2 c_4} - \frac{6 \mu c_2^2 c_4 \left( 1 + \coth \left[ \frac{\sqrt{c_2}}{2} (x - \eta t) \right] \right) ^2 }{c_3^2} \\ + 3 \mu c_2 \left( 1 + \coth \left[ \frac{\sqrt{c_2}}{2} (x - \eta t) \right] - \frac{ \sqrt{c_2 c_4} \, {{\,\textrm{csch}\,}}^2\left[ \frac{\sqrt{c_2}}{2} (x - \eta t) \right] }{c_3} \right) , \end{aligned} \end{aligned}$$

describes a singular soliton.

From Subcase [5.4] the following explicit solutions are obtained:

${\textbf {[5.4.1]}}$ If $c_2> 0$, $c_4> 0$, and $c_2 \ne \frac{c_3^2}{4 c_4}$, then

42 $$\begin{aligned} u_{5.4.1}(x, t) = -\frac{3 \mu c_2 \left( -c_3^2 + 4 c_2 c_4 \right) }{\left( c_3 \cosh \left[ \frac{\sqrt{c_2}}{2} (x - \eta t) \right] - 2 \sqrt{c_2 c_4} \sinh \left[ \frac{\sqrt{c_2}}{2} (x - \eta t) \right] \right) ^2}, \end{aligned}$$

yields a hyperbolic.

${\textbf {[5.4.2]}}$ If $c_2 < 0$, $c_4> 0$, and $c_2 \ne \frac{c_3^2}{4 c_4}$, then

43 $$\begin{aligned} u_{5.4.2}(x, t) = -\frac{3 \mu c_2 \left( -c_3^2 + 4 c_2 c_4 \right) }{\left( c_3 \cos \left[ \frac{\sqrt{-c_2}}{2} (x - \eta t) \right] + 2 \sqrt{-c_2 c_4} \sin \left[ \frac{\sqrt{-c_2}}{2} (x - \eta t) \right] \right) ^2}, \end{aligned}$$

yields a periodic.

From Subcase [5.5] the following explicit solutions are obtained:

${\textbf {[5.5.1]}}$ If $c_2> 0$, $c_4> 0$, and $c_2 \ne \frac{c_3^2}{4 c_4}$, then

44 $$\begin{aligned} \begin{aligned} u_{5.5.1}(x, t)&= -\frac{\mu c_2 {{\,\textrm{sech}\,}}^{2}\left[ \frac{(x - \eta t) \sqrt{c_{2}}}{2} \right] \left( { c_{3}^{2} \left( -2 + \cosh \left[ (x - \eta t) \sqrt{c_{2}} \right] \right) + 4 c_{2} c_{4} \left( 2 + \cosh \left[ (x - \eta t) \sqrt{c_{2}} \right] \right) }{- 4 c_{3} \sqrt{c_{2} c_{4}} \sinh \left[ (x - \eta t) \sqrt{c_{2}} \right] } \right) }{\left( c_{3} - 2 \sqrt{c_{2} c_{4}} \tanh \left[ \frac{(x - \eta t) \sqrt{c_{2}}}{2} \right] \right) ^{2}}, \end{aligned} \end{aligned}$$

yields a hyperbolic.

${\textbf {[5.5.2]}}$ If $c_2 < 0$, $c_4> 0$, and $c_2 \ne \frac{c_3^2}{4 c_4}$, then

45 $$\begin{aligned} \begin{aligned} u_{5.5.2}(x, t)&= -\frac{\mu c_2 \sec ^{2}\left[ \frac{(x - \eta t) \sqrt{-c_{2}}}{2} \right] \left( { c_{3}^{2} \left( -2 + \cos \left[ (x - \eta t) \sqrt{-c_{2}} \right] \right) + 4 c_{2} c_{4} \left( 2 + \cos \left[ (x - \eta t) \sqrt{-c_{2}} \right] \right) }{+ 4 c_{3} \sqrt{-c_{2} c_{4}} \sin \left[ (x - \eta t) \sqrt{-c_{2}} \right] } \right) }{\left( c_{3} + 2 \sqrt{-c_{2} c_{4}} \tan \left[ \frac{(x - \eta t) \sqrt{-c_{2}}}{2} \right] \right) ^{2}}, \end{aligned} \end{aligned}$$

describes a singular periodic.

**Case Study 6:** $$c_2 = c_4 = c_5 = 0$$

In this family the even-powered interior terms ($c_2$, $c_4$) and the sextic term are suppressed, leaving $\mathcal {W}' = \sqrt{c_0 + c_1 \mathcal {W} + c_3\mathcal {W}^3}$, a cubic polynomial radicand. This is the defining auxiliary equation of the Weierstrass elliptic function $\wp (z; g_2, g_3)$, whose solution is doubly periodic in the complex plane. The solution obtained in this case therefore represents a genuinely elliptic wave structure, with lattice invariants expressible directly in terms of $c_0$, $c_1$, and $c_3$. One parameter family is obtained.

$${\textbf {[6.1]}}$$ $$p_0 = \frac{i \sqrt{c_1} \sqrt{c_3}}{2 \sqrt{3} \sqrt{c_0}}, \quad p_1 = p_2 = 0, \quad q_1 = \frac{c_1}{2 \sqrt{c_0}}, \quad q_2 = \sqrt{c_0}, \quad r_2 = s_1 = 0, \quad s_2 = 1, \quad \mu = -\frac{1}{6 \sqrt{c_0}}, \quad \eta = -\frac{i \left( -i + \sqrt{3} \sqrt{c_1} \sqrt{c_3}\right) }{6 \sqrt{c_0}}.$$

From Subcase [6.1] the following explicit solution are obtained:

${\textbf {[6.1.1]}}$ If $c_3> 0$, $c_0 \ne 0$, and $c_1 \ne 0$, then

46 $$\begin{aligned} \begin{aligned} u_{6.1.1}(x, t) = \frac{6 c_0 + 3 c_1 \,\, \wp \left[ \frac{\sqrt{c_3}}{2} (x - \eta t); g_1, g_2 \right] + i \sqrt{3} \sqrt{c_1 c_3} \,\, \wp ^2\left[ \frac{\sqrt{c_3}}{2} (x - \eta t); g_1, g_2 \right] }{6 \sqrt{c_0} \,\, \wp ^2\left[ \frac{\sqrt{c_3}}{2} (x - \eta t); g_1, g_2 \right] } \\ + \frac{3 \sqrt{c_0 c_3} \,\, \wp '\left[ \frac{\sqrt{c_3}}{2} (x - \eta t); g_1, g_2 \right] }{6 \sqrt{c_0} \,\, \wp ^2\left[ \frac{\sqrt{c_3}}{2} (x - \eta t); g_1, g_2 \right] }, \end{aligned} \end{aligned}$$

is a Weierstrass elliptic solution, where $\wp (z; g_1, g_2)$ is the Weierstrass elliptic function with invariants $g_1 = -\frac{4 c_1}{c_3}$ and $g_2 = -\frac{4 c_0}{c_3}$.

**Case Study 7:** $$c_1 = 0$$, $$c_3 = 0$$

Suppressing the odd-powered terms ($c_1 = c_3 = 0$) renders the auxiliary equation symmetric in $\mathcal {W}$: $\mathcal {W}' = \sqrt{c_0 + c_2\mathcal {W}^2 + c_4\mathcal {W}^4 + c_5\mathcal {W}^6}$. The sextic coefficient $c_5$ is retained here, allowing for a richer algebraic structure. The resulting solutions take a hyperbolic or trigonometric form involving $\cosh$ and $\cos$ functions weighted by a parameter combination $\sqrt{c_4^2 - 4c_2 c_5}$, which governs the modulation structure. One consistent parameter set is found.

$${\textbf {[7.1]}}$$ $$p_0 = \frac{1}{2} \left( -\eta - 4 \eta c_2 \right) , \quad p_1 = p_2 = q_1 = 0, \quad q_2 = \frac{\eta - 16 \eta c_2^2}{8 c_4}, \quad r_2 = s_1 = s_2 = 0, \quad \mu = \frac{\eta }{2}, \quad c_0 = \frac{-1 + 16 c_2^2}{48 c_4}.$$

From Subcase [7.1] the following explicit solutions are obtained:

$${\textbf {[7.1.1]}}$$ If $$c_2> 0$$, then

47 $$\begin{aligned} \begin{aligned} u_{7.1.1}(x, t) = -\frac{\eta (1 + 4 c_2)}{16 c_2 c_4} \Big ( (1 + 4 c_2) c_4 + \cosh \left[ 2 \sqrt{c_2} (x - \eta t) \right] (-1 + 4 c_2) \sqrt{c_4^2 - 4 c_2 c_5} \Big ), \end{aligned} \end{aligned}$$

yields a hyperbolic.

$${\textbf {[7.1.2]}}$$ If $$c_2 < 0$$, then

48 $$\begin{aligned} \begin{aligned} u_{7.1.2}(x, t) = -\frac{\eta (1 + 4 c_2)}{16 c_2 c_4} \Big ( (-1 + 12 c_2) c_4 + \cos \left[ 2 \sqrt{-c_2} (x - \eta t) \right] (-1 + 4 c_2) \sqrt{c_4^2 - 4 c_2 c_5} \Big ), \end{aligned} \end{aligned}$$

yields a periodic.

**Case Study 8:** $$c_1 = 0$$, $$c_3 = 0$$, $$c_5 = 0$$

This is the most general even-symmetric case with $c_5$ also set to zero: $\mathcal {W}' = \sqrt{c_0 + c_2\mathcal {W}^2 + c_4\mathcal {W}^4}$. This auxiliary equation is the standard quartic elliptic differential equation whose general solution is expressed in terms of Jacobi elliptic functions $\text {sn}$, $\text {cn}$, $\text {dn}$ and their combinations, parametrized by the elliptic modulus $m \in [0,1]$. The limiting cases $m \rightarrow 0$ and $m \rightarrow 1$ recover trigonometric and hyperbolic (soliton) solutions respectively, providing a continuous family of solutions interpolating between periodic and localized regimes. This case yields the largest number of parameter families (five) and the most extensive set of Jacobi elliptic solutions in the present study.

$${\textbf {[8.1]}}$$ $$p_0 = 4 \left( -\mu c_2 - \sqrt{\mu ^2 \left( c_2^2 - 3 c_0 c_4 \right) } \right) , \quad p_1 = 0, \quad p_2 = -12 \mu c_4, \quad q_1 = q_2 = r_2 = s_1 = s_2 = 0, \quad \eta = \mu + 4 \sqrt{\mu ^2 \left( c_2^2 - 3 c_0 c_4 \right) }.$$

$${\textbf {[8.2]}}$$ $$p_0 = 4 \left( -\mu c_2 - \sqrt{\mu ^2 \left( c_2^2 - 3 c_0 c_4 \right) } \right) , \quad p_1 = p_2 = q_1 = 0, \quad q_2 = -12 \mu c_0, \quad r_2 = s_1 = s_2 = 0, \quad \eta = \mu + 4 \sqrt{\mu ^2 \left( c_2^2 - 3 c_0 c_4 \right) }.$$

$${\textbf {[8.3]}}$$ $$p_0 = -\mu c_2 + \sqrt{\mu ^2 \left( c_2^2 + 12 c_0 c_4 \right) }, \quad p_1 = p_2 = q_1 = 0, \quad q_2 = -6 \mu c_0, \quad r_2 = s_1 = 0, \quad s_2 = 6 \mu \sqrt{c_0}, \quad \eta = \mu - \sqrt{\mu ^2 \left( c_2^2 + 12 c_0 c_4 \right) }.$$

$${\textbf {[8.4]}}$$ $$p_0 = 4 \left( -\mu c_2 - \sqrt{\mu ^2 \left( c_2^2 + 12 c_0 c_4 \right) } \right) , \quad p_1 = 0, \quad p_2 = -12 \mu c_4, \quad q_1 = 0, \quad q_2 = -12 \mu c_0, \quad r_2 = s_1 = s_2 = 0, \quad \eta = \mu + 4 \sqrt{\mu ^2 \left( c_2^2 + 12 c_0 c_4 \right) }.$$

$${\textbf {[8.5]}}$$ $$p_0 = -\mu c_2 + \sqrt{\mu ^2 \left( c_2^2 + 12 c_0 c_4 \right) }, \quad p_1 = 0, \quad p_2 = -6 \mu c_4, \quad q_1 = q_2 = 0, \quad r_2 = 6 \mu \sqrt{c_4}, \quad s_1 = s_2 = 0, \quad \eta = \mu - \sqrt{\mu ^2 \left( c_2^2 + 12 c_0 c_4 \right) }.$$

From Subcase [8.1] the following explicit solutions are obtained:

${\textbf {[8.1.1]}}$ If $0 \le m \le 1$, $c_0 = 1$, $c_2 = -(1 + m^2)$, and $c_4 = m^2$, then

49 $$\begin{aligned} \begin{aligned} u_{8.1.1}(x, t) = -4 \left( - \mu ( 1 + m^2 ) + \sqrt{(1 - m^2 + m^4) \mu ^2} + 3 m^2 \mu \, {{\,\textrm{cd}\,}}^2\left[ (x - \eta t) \right] \right) , \end{aligned} \end{aligned}$$

represents a Jacobi elliptic.

${\textbf {[8.1.2]}}$ If $0 \le m \le 1$, $c_0 = m^2 - 1$, $c_2 = -m^2 + 2$, and $c_4 = -1$, then

50 $$\begin{aligned} \begin{aligned} u_{8.1.2}(x, t) = 4 \left( (-2 + m^2) \mu - \sqrt{(1 - m^2 + m^4) \mu ^2} + 12 \mu \, {{\,\textrm{dn}\,}}^2\left[ (x - \eta t) \right] \right) , \end{aligned} \end{aligned}$$

represents a Jacobi elliptic.

If $$m = 1$$, then

51 $$\begin{aligned} u_{8.1.2a}(x, t) = -4 \left( \mu + \sqrt{\mu ^2} - 3 \mu {{\,\textrm{sech}\,}}^2\left[ (x - \eta t) \right] \right) , \end{aligned}$$

describes a bright soliton.

${\textbf {[8.1.3]}}$ If $0 \le m \le 1$, $c_0 = -m^2$, $c_2 = 2 m^2 - 1$, and $c_4 = -m^2 + 1$, then

52 $$\begin{aligned} \begin{aligned} u_{8.1.3}(x, t) = -4 \left( (-1 + 2 m^2) \mu + \sqrt{(1 - m^2 + m^4) \mu ^2} - 3 (-1 + m^2) \mu \, {{\,\textrm{nc}\,}}^2\left[ (x - \eta t) \right] \right) , \end{aligned} \end{aligned}$$

represents a Jacobi elliptic.

If $$m = 0$$, then

53 $$\begin{aligned} u_{8.1.3a}(x, t) = -4 \left( -\mu + \sqrt{\mu ^2} + 3 \mu \sec ^2\left[ (x - \eta t) \right] \right) , \end{aligned}$$

describes a singular periodic.

${\textbf {[8.1.4]}}$ If $0 < m \le 1$, $c_0 = m^2 - 2 m^3 + m^4$, $c_2 = -\frac{4}{m}$, and $c_4 = -1 + 6 m - m^2$, then

54 $$\begin{aligned} \begin{aligned} u_{8.1.4}(x, t) = \frac{4}{m} \left( 4 \mu - \sqrt{(16 + 3 m^4 - 24 m^5 + 42 m^6 - 24 m^7 + 3 m^8) \mu ^2} \right. \\ \left. + \frac{3 m^3 (1 - 6 m + m^2) \mu \, {{\,\textrm{cn}\,}}^2\left[ (x - \eta t) \right] \, {{\,\textrm{dn}\,}}^2\left[ (x - \eta t) \right] }{(1 + m \, {{\,\textrm{sn}\,}}^2\left[ (x - \eta t) \right] )^2} \right) , \end{aligned} \end{aligned}$$

represents a Jacobi elliptic.

If $$m = 1$$, then

55 $$\begin{aligned} u_{8.1.4a}(x, t) = -16 \left( -\mu + \sqrt{\mu ^2} + 3 \mu {{\,\textrm{sech}\,}}^2\left[ 2 (x - \eta t) \right] \right) , \end{aligned}$$

describes a bright soliton.

${\textbf {[8.1.5]}}$ If $0 \le m \le 1$, $c_0 = \frac{1}{4}$, $c_2 = \frac{1}{2} m^2 - 1$, and $c_4 = \frac{m^4}{4}$, then

56 $$\begin{aligned} \begin{aligned} u_{8.1.5}(x, t) = -2 \left( (-2 + m^2) \mu + \sqrt{(16 - 16 m^2 + m^4) \mu ^2} + \frac{3 m^4 \mu \, {{\,\textrm{sn}\,}}^2\left[ (x - \eta t) \right] }{(1 + \, {{\,\textrm{dn}\,}}\left[ (x - \eta t) \right] )^2} \right) , \end{aligned} \end{aligned}$$

represents a Jacobi elliptic.

If $$m = 1$$, then

57 $$\begin{aligned} u_{8.1.5a}(x, t) = 2 \mu - \sqrt{\mu ^2} - 3 \mu \tanh ^2\left[ \frac{(x - \eta t)}{2} \right] , \end{aligned}$$

describes a dark soliton.

From Subcase [8.2] the following explicit solutions are obtained:

${\textbf {[8.2.1]}}$ If $0 \le m \le 1$, $c_0 = 1$, $c_2 = -(1 + m^2)$, and $c_4 = m^2$, then

58 $$\begin{aligned} \begin{aligned} u_{8.2.1}(x, t) = 4 \left( \mu + m^2 \mu - \sqrt{(1 - m^2 + m^4) \mu ^2} - \frac{3 \mu }{\, {{\,\textrm{cn}\,}}^2\left[ (x - \eta t) \right] \, {{\,\textrm{dn}\,}}^2\left[ (x - \eta t) \right] } \right) , \end{aligned} \end{aligned}$$

represents a Jacobi elliptic.

${\textbf {[8.2.2]}}$ If $0 \le m \le 1$, $c_0 = m^2 - 1$, $c_2 = -m^2 + 2$, and $c_4 = -1$, then

59 $$\begin{aligned} \begin{aligned} u_{8.2.2}(x, t) = 4 \left( (-2 + m^2) \mu - \sqrt{(1 - m^2 + m^4) \mu ^2} - \frac{3 (-1 + m^2) \mu }{\, {{\,\textrm{dn}\,}}^2\left[ (x - \eta t) \right] } \right) , \end{aligned} \end{aligned}$$

represents a Jacobi elliptic.

${\textbf {[8.2.3]}}$ If $0 \le m \le 1$, $c_0 = -m^2$, $c_2 = 2 m^2 - 1$, and $c_4 = -m^2 + 1$, then

60 $$\begin{aligned} \begin{aligned} u_{8.2.3}(x, t) = 4 \left( \mu - 2 m^2 \mu - \sqrt{(1 - m^2 + m^4) \mu ^2} + \frac{3 m^2 \mu }{\, {{\,\textrm{nc}\,}}^2\left[ (x - \eta t) \right] } \right) , \end{aligned} \end{aligned}$$

represents a Jacobi elliptic.

${\textbf {[8.2.4]}}$ If $0 \le m \le 1$, $c_0 = -1$, $c_2 = -m^2 + 2$, and $c_4 = m^2 - 1$, then

61 $$\begin{aligned} \begin{aligned} u_{8.2.4}(x, t) = 4 \left( (-2 + m^2) \mu - \sqrt{(1 - m^2 + m^4) \mu ^2} + \frac{3 \mu }{\, {{\,\textrm{nd}\,}}^2\left[ (x - \eta t) \right] } \right) , \end{aligned} \end{aligned}$$

represents a Jacobi elliptic.

${\textbf {[8.2.5]}}$ If $0 < m \le 1$, $c_0 = m^2 - 2 m^3 + m^4$, $c_2 = -\frac{4}{m}$, and $c_4 = -1 + 6 m - m^2$, then

62 $$\begin{aligned} \begin{aligned} u_{8.2.5}(x, t) = -\frac{4}{m} \left( -4 \mu + \sqrt{(16 + 3 m^4 - 24 m^5 + 42 m^6 - 24 m^7 + 3 m^8) \mu ^2} \right. \\ \left. + \frac{12 (-1 + m)^2 \mu (1 + m \, {{\,\textrm{sn}\,}}^2\left[ (x - \eta t) \right] )^2}{\, {{\,\textrm{cn}\,}}^2\left[ (x - \eta t) \right] \, {{\,\textrm{dn}\,}}^2\left[ (x - \eta t) \right] } \right) , \end{aligned} \end{aligned}$$

represents a Jacobi elliptic.

${\textbf {[8.2.6]}}$ If $0 \le m \le 1$, $c_0 = \frac{1}{4}$, $c_2 = \frac{1}{2} m^2 - 1$, and $c_4 = \frac{m^4}{4}$, then

63 $$\begin{aligned} \begin{aligned} u_{8.2.6}(x, t) = -\frac{3 \mu + 6 \mu \, {{\,\textrm{dn}\,}}\left[ (x - \eta t) \right] + 3 \mu \, {{\,\textrm{dn}\,}}^2\left[ (x - \eta t) \right] }{\, {{\,\textrm{sn}\,}}^2\left[ (x - \eta t) \right] } \\ - \frac{\left( 2 (-2 + m^2) \mu + \sqrt{(16 - 16 m^2 + m^4) \mu ^2} \right) \, {{\,\textrm{sn}\,}}^2\left[ (x - \eta t) \right] }{\, {{\,\textrm{sn}\,}}^2\left[ (x - \eta t) \right] }, \end{aligned} \end{aligned}$$

represents a Jacobi elliptic.

When the elliptic modulus satisfies $m = 0$ or $m = 1$, the Jacobi elliptic solutions degenerate into elementary forms, yielding the following singular periodic and singular soliton profiles:

64 $$\begin{aligned} u_{8.2.6a}(x, t) = -4 \left( -\mu + \sqrt{\mu ^2} + 3 \mu \csc ^2\left[ (x - \eta t) \right] \right) , \end{aligned}$$

or

65 $$\begin{aligned} u_{8.2.6b}(x, t) = 2 \mu - \sqrt{\mu ^2} - 3 \mu \coth ^2\left[ \frac{x - \eta t}{2} \right] . \end{aligned}$$

From Subcase [8.3] the following explicit solutions are obtained:

${\textbf {[8.3.1]}}$ If $0 \le m \le 1$, $c_0 = 1$, $c_2 = -(1 + m^2)$, and $c_4 = m^2$, then

66 $$\begin{aligned} \begin{aligned} u_{8.3.1}(x, t) = \mu + m^2 \mu + \sqrt{(1 + 14 m^2 + m^4) \mu ^2} + \frac{6 \mu \left( -1 + (-1 + m) \, {{\,\textrm{nd}\,}}\left[ (x - \eta t) \right] \, {{\,\textrm{sd}\,}}\left[ (x - \eta t) \right] \right) }{\, {{\,\textrm{cn}\,}}^2 \left[ (x - \eta t) \right] \, {{\,\textrm{dn}\,}}^2 \left[ (x - \eta t) \right] }, \end{aligned} \end{aligned}$$

represents a Jacobi elliptic.

${\textbf {[8.3.2]}}$ If $0 \le m \le 1$, $c_0 = m^2 - 1$, $c_2 = -m^2 + 2$, and $c_4 = -1$, then

67 $$\begin{aligned} \begin{aligned} u_{8.3.2}(x, t) = (-2 + m^2) \mu + \sqrt{(16 - 16 m^2 + m^4) \mu ^2} \\ - \frac{6 \mu \left( -1 + m^2 + m \sqrt{-1 + m^2} \, {{\,\textrm{cn}\,}}\left[ (x - \eta t) \right] \, {{\,\textrm{sn}\,}}\left[ (x - \eta t) \right] \right) }{\, {{\,\textrm{dn}\,}}^2\left[ (x - \eta t) \right] }, \end{aligned} \end{aligned}$$

represents a Jacobi elliptic.

${\textbf {[8.3.3]}}$ If $0 \le m \le 1$, $c_0 = \frac{1}{4}$, $c_2 = \frac{1}{2} m^2 - 1$, and $c_4 = \frac{m^4}{4}$, then

68 $$\begin{aligned} \begin{aligned} u_{8.3.3}(x, t) = \frac{1}{2 \, {{\,\textrm{sn}\,}}^2\left[ (x - \eta t) \right] } \Big ( -3 \mu + 6 \mu (-1 + \, {{\,\textrm{cn}\,}}\left[ (x - \eta t) \right] ) \, {{\,\textrm{dn}\,}}\left[ (x - \eta t) \right] \\ + 3 \mu (-1 + 2 \, {{\,\textrm{cn}\,}}\left[ (x - \eta t) \right] ) \, {{\,\textrm{dn}\,}}^2\left[ (x - \eta t) \right] \\ + \left( 2 \mu - m^2 \mu + 2 \sqrt{(1 - m^2 + m^4) \mu ^2} + 6 m \mu \, {{\,\textrm{cn}\,}}\left[ (x - \eta t) \right] \right) \, {{\,\textrm{sn}\,}}^2\left[ (x - \eta t) \right] \Big ), \end{aligned} \end{aligned}$$

represents a Jacobi elliptic.

From Subcase [8.4] the following explicit solutions are obtained:

${\textbf {[8.4.1]}}$ If $0 \le m \le 1$, $c_0 = 1$, $c_2 = -(1 + m^2)$, and $c_4 = m^2$, then

69 $$\begin{aligned} \begin{aligned} u_{8.4.1}(x, t) = 4 \left( \mu + m^2 \mu - \sqrt{(1 + 14 m^2 + m^4) \mu ^2} - \frac{3 \mu }{\, {{\,\textrm{cn}\,}}^2\left[ (x - \eta t) \right] \, {{\,\textrm{dn}\,}}^2\left[ (x - \eta t) \right] } \right. \\ - 3 m^2 \mu \, {{\,\textrm{cn}\,}}^2\left[ (x - \eta t) \right] \, {{\,\textrm{dn}\,}}^2\left[ (x - \eta t) \right] \bigg ), \end{aligned} \end{aligned}$$

represents a Jacobi elliptic.

${\textbf {[8.4.2]}}$ If $0 \le m \le 1$, $c_0 = m^2 - 1$, $c_2 = -m^2 + 2$, and $c_4 = -1$, then

70 $$\begin{aligned} \begin{aligned} u_{8.4.2}(x, t) = 4 \left( (-2 + m^2) \mu - \sqrt{(16 - 16 m^2 + m^4) \mu ^2} - \frac{3 (-1 + m^2) \mu }{\, {{\,\textrm{dn}\,}}^2\left[ (x - \eta t) \right] } + 3 \mu \, {{\,\textrm{dn}\,}}^2\left[ (x - \eta t) \right] \right) , \end{aligned} \end{aligned}$$

represents a Jacobi elliptic.

${\textbf {[8.4.3]}}$ If $0 \le m \le 1$, $c_0 = -m^2$, $c_2 = 2 m^2 - 1$, and $c_4 = -m^2 + 1$, then

71 $$\begin{aligned} \begin{aligned} u_{8.4.3}(x, t) = (4 - 8 m^2) \mu - 4 \sqrt{(1 - 16 m^2 + 16 m^4) \mu ^2} + \frac{12 m^2 \mu }{\, {{\,\textrm{nc}\,}}^2\left[ (x - \eta t) \right] } \\ + 12 (-1 + m^2) \mu \, {{\,\textrm{nc}\,}}^2\left[ (x - \eta t) \right] , \end{aligned} \end{aligned}$$

represents a Jacobi elliptic.

When the elliptic modulus satisfies $m = 0$ or $m = 1$, the Jacobi elliptic solutions degenerate into elementary forms, yielding the following singular periodic and bright soliton profiles:

72 $$\begin{aligned} u_{8.4.3a}(x, t) = -4 \left( -\mu + \sqrt{\mu ^2} + 3 \mu \sec ^2\left[ (x - \eta t) \right] \right) , \end{aligned}$$

or

73 $$\begin{aligned} u_{8.4.3b}(x, t) = -4 \left( \mu + \sqrt{\mu ^2} - 3 \mu {{\,\textrm{sech}\,}}^2\left[ (x - \eta t) \right] \right) . \end{aligned}$$

${\textbf {[8.4.4]}}$ If $0 \le m \le 1$, $c_0 = -1$, $c_2 = -m^2 + 2$, and $c_4 = m^2 - 1$, then

74 $$\begin{aligned} \begin{aligned} u_{8.4.4}(x, t) = 4 \left( (-2 + m^2) \mu - \sqrt{(16 - 16 m^2 + m^4) \mu ^2} + \frac{3 \mu }{\, {{\,\textrm{nd}\,}}^2\left[ (x - \eta t) \right] } \right. \\ \left. - 3 (-1 + m^2) \mu \, {{\,\textrm{nd}\,}}^2\left[ (x - \eta t) \right] \right) , \end{aligned} \end{aligned}$$

represents a Jacobi elliptic.

${\textbf {[8.4.5]}}$ If $0 < m \le 1$, $c_0 = m^2 - 2 m^3 + m^4$, $c_2 = -\frac{4}{m}$, and $c_4 = -1 + 6 m - m^2$, then

75 $$\begin{aligned} \begin{aligned} u_{8.4.5}(x, t) = 4 \left( \frac{4 \mu }{m} - 2 \sqrt{- \frac{(-4 + 3 m^4 - 24 m^5 + 42 m^6 - 24 m^7 + 3 m^8) \mu ^2}{m^2}} \right. \\ \left. + \frac{3 m^2 (1 - 6 m + m^2) \mu \, {{\,\textrm{cn}\,}}^2\left[ (x - \eta t) \right] \, {{\,\textrm{dn}\,}}^2\left[ (x - \eta t) \right] }{(1 + m \, {{\,\textrm{sn}\,}}^2\left[ (x - \eta t) \right] )^2} - \frac{3 (-1 + m)^2 \mu (1 + m \, {{\,\textrm{sn}\,}}^2\left[ (x - \eta t) \right] )^2}{\, {{\,\textrm{cn}\,}}^2\left[ (x - \eta t) \right] \, {{\,\textrm{dn}\,}}^2\left[ (x - \eta t) \right] } \right) , \end{aligned} \end{aligned}$$

represents a Jacobi elliptic.

If $$m = 1$$, then

76 $$\begin{aligned} u_{8.4.5a}(x, t) = -16 \left( -\mu + \sqrt{\mu ^2} + 3 \mu {{\,\textrm{sech}\,}}^2\left[ 2 (x - \eta t) \right] \right) , \end{aligned}$$

describes a bright soliton.

${\textbf {[8.4.6]}}$ If $0 \le m \le 1$, $c_0 = \frac{1}{4}$, $c_2 = \frac{1}{2} m^2 - 1$, and $c_4 = \frac{m^4}{4}$, then

77 $$\begin{aligned} \begin{aligned} u_{8.4.6}(x, t) = -2 \left( (-2 + m^2) \mu + 4 \sqrt{(1 - m^2 + m^4) \mu ^2} \right. \\ \left. + \frac{3 \mu (1 + \, {{\,\textrm{dn}\,}}\left[ (x - \eta t) \right] )^2}{\, {{\,\textrm{sn}\,}}^2\left[ (x - \eta t) \right] } + \frac{3 m^4 \mu \, {{\,\textrm{sn}\,}}^2\left[ (x - \eta t) \right] }{(1 + \, {{\,\textrm{dn}\,}}\left[ (x - \eta t) \right] )^2} \right) , \end{aligned} \end{aligned}$$

represents a Jacobi elliptic.

When the elliptic modulus satisfies $m = 0$ or $m = 1$, the Jacobi elliptic solutions degenerate into elementary forms, yielding the following singular periodic and combo singular-dark soliton profiles:

78 $$\begin{aligned} u_{8.4.6a}(x, t) = -4 \left( -\mu + \sqrt{\mu ^2} + 3 \mu \csc ^2\left[ (x - \eta t) \right] \right) , \end{aligned}$$

or

79 $$\begin{aligned} u_{8.4.6b}(x, t) = 2 \mu - 4 \sqrt{\mu ^2} - 3 \mu \coth ^2\left[ \frac{x - \eta t}{2} \right] - 3 \mu \tanh ^2\left[ \frac{x - \eta t}{2} \right] . \end{aligned}$$

From Subcase [8.5] the following explicit solutions are obtained:

${\textbf {[8.5.1]}}$ If $0 \le m \le 1$, $c_0 = 1$, $c_2 = -(1 + m^2)$, and $c_4 = m^2$, then

80 $$\begin{aligned} \begin{aligned} u_{8.5.1}(x, t) = \mu + m^2 \mu + \sqrt{(1 + 14 m^2 + m^4) \mu ^2} - 6 m^2 \mu \, {{\,\textrm{cn}\,}}^2\left[ (x - \eta t) \right] \, {{\,\textrm{dn}\,}}^2\left[ (x - \eta t) \right] \\ + 6 (-1 + m) m \mu \, {{\,\textrm{nd}\,}}\left[ (x - \eta t) \right] \, {{\,\textrm{sd}\,}}\left[ (x - \eta t) \right] , \end{aligned} \end{aligned}$$

represents a Jacobi elliptic.

${\textbf {[8.5.2]}}$ If $0 \le m \le 1$, $c_0 = m^2 - 1$, $c_2 = -m^2 + 2$, and $c_4 = -1$, then

81 $$\begin{aligned} \begin{aligned} u_{8.5.2}(x, t) = -2 \mu + m^2 \mu + \sqrt{(16 - 16 m^2 + m^4) \mu ^2} + 6 \mu \, {{\,\textrm{dn}\,}}^2\left[ (x - \eta t) \right] \\ - 6 i m \mu \, {{\,\textrm{cn}\,}}\left[ (x - \eta t) \right] \, {{\,\textrm{sn}\,}}\left[ (x - \eta t) \right] , \end{aligned} \end{aligned}$$

represents a Jacobi elliptic.

${\textbf {[8.5.3]}}$ If $0 \le m \le 1$, $c_0 = -m^2$, $c_2 = 2 m^2 - 1$, and $c_4 = -m^2 + 1$, then

82 $$\begin{aligned} \begin{aligned} u_{8.5.3}(x, t) = \mu - 2 m^2 \mu + \sqrt{(1 - 16 m^2 + 16 m^4) \mu ^2} + 6 (-1 + m^2) \mu \, {{\,\textrm{nc}\,}}^2\left[ (x - \eta t) \right] \\ + 6 \sqrt{1 - m^2} \mu \, {{\,\textrm{dc}\,}}\left[ (x - \eta t) \right] \, \, {{\,\textrm{sn}\,}}\left[ (x - \eta t) \right] , \end{aligned} \end{aligned}$$

represents a Jacobi elliptic.

${\textbf {[8.5.4]}}$ If $0 \le m \le 1$, $c_0 = -1$, $c_2 = -m^2 + 2$, and $c_4 = m^2 - 1$, then

83 $$\begin{aligned} \begin{aligned} u_{8.5.4}(x, t) = -2 \mu + m^2 \mu + \sqrt{(16 - 16 m^2 + m^4) \mu ^2} - 6 (-1 + m^2) \mu \, {{\,\textrm{nd}\,}}^2\left[ (x - \eta t) \right] \\ + 6 m \sqrt{-1 + m^2} \mu \, {{\,\textrm{cn}\,}}\left[ (x - \eta t) \right] \, {{\,\textrm{dn}\,}}\left[ (x - \eta t) \right] , \end{aligned} \end{aligned}$$

represents a Jacobi elliptic.

${\textbf {[8.5.5]}}$ If $0 < m \le 1$, $c_0 = m^2 - 2 m^3 + m^4$, $c_2 = -\frac{4}{m}$, and $c_4 = -1 + 6 m - m^2$, then

84 $$\begin{aligned} \begin{aligned}&u_{8.5.5}(x, t) = \frac{4 \mu }{m} + 2 \sqrt{\left( \frac{4}{m^2} - 3 (-1 + m)^2 m^2 (1 - 6 m + m^2) \right) \mu ^2} \\&+ \frac{6 m^2 (1 - 6 m + m^2) \mu \, {{\,\textrm{cn}\,}}^2\left[ (x - \eta t) \right] \, {{\,\textrm{dn}\,}}^2\left[ (x - \eta t) \right] }{(1 + m \, {{\,\textrm{sn}\,}}^2\left[ (x - \eta t) \right] )^2} \\&- \frac{6 m \sqrt{-1 + 6 m - m^2} \mu \, {{\,\textrm{sn}\,}}\left[ (x - \eta t) \right] }{(1 + m \, {{\,\textrm{sn}\,}}^2\left[ (x - \eta t) \right] )^2} \bigg ( \, {{\,\textrm{dn}\,}}^2\left[ (x - \eta t) \right] (1 + m \, {{\,\textrm{sn}\,}}^2\left[ (x - \eta t) \right] ) \\&+ m \, {{\,\textrm{cn}\,}}^2\left[ (x - \eta t) \right] (1 + 2 \, {{\,\textrm{dn}\,}}^2\left[ (x - \eta t) \right] + m \, {{\,\textrm{sn}\,}}^2\left[ (x - \eta t) \right] ) \bigg ), \end{aligned} \end{aligned}$$

represents a Jacobi elliptic.

If $$m = 1$$, then

85 $$\begin{aligned} \begin{aligned} u_{8.5.5a}(x, t) = 2 {{\,\textrm{sech}\,}}^2\left[ 2 (x - \eta t) \right] \left( -11 \mu + \sqrt{\mu ^2} + (\mu + \sqrt{\mu ^2}) \cosh \left[ 4 (x - \eta t) \right] - 12 \mu \sinh \left[ 2 (x - \eta t) \right] \right) , \end{aligned} \end{aligned}$$

yields a hyperbolic.

${\textbf {[8.5.6]}}$ If $0 \le m \le 1$, $c_0 = \frac{1}{4}$, $c_2 = \frac{1}{2} m^2 - 1$, and $c_4 = \frac{m^4}{4}$, then

86 $$\begin{aligned} \begin{aligned} u_{8.5.6}(x, t) = \mu - \frac{m^2 \mu }{2} + \sqrt{(1 - m^2 + m^4) \mu ^2} - \frac{3 m^4 \mu \, {{\,\textrm{sn}\,}}^2\left[ (x - \eta t) \right] }{2 (1 + \, {{\,\textrm{dn}\,}}\left[ (x - \eta t) \right] )^2} \\ + \frac{3 m^2 \mu \, {{\,\textrm{cn}\,}}\left[ (x - \eta t) \right] \left( \, {{\,\textrm{dn}\,}}\left[ (x - \eta t) \right] + \, {{\,\textrm{dn}\,}}^2\left[ (x - \eta t) \right] + m \, {{\,\textrm{sn}\,}}^2\left[ (x - \eta t) \right] \right) }{(1 + \, {{\,\textrm{dn}\,}}\left[ (x - \eta t) \right] )^2}, \end{aligned} \end{aligned}$$

represents a Jacobi elliptic.

## An examination of instability in the suggested model

This section conducts a linear stability analysis of Eq. (1). Before proceeding with the linear stability analysis, we first verify that a constant state $u = \mathcal {R}$ (where $\mathcal {R}$ is an arbitrary real constant) constitutes an exact solution of Eq. (1). Substituting $u = \mathcal {R} = \text {const}$ into Eq. (1), all spatial and temporal derivatives vanish identically, yielding $0 = 0$. Thus, any constant $\mathcal {R}$ is an admissible background solution. We now perform a linear stability analysis about this constant background state by introducing small perturbations of the form

87 $$\begin{aligned} u(x,t) = \mathcal {R} + \gamma \mathcal {P}(x,t), \end{aligned}$$

where $\mathcal {R}$ is the constant background solution, $\mathcal {P}(x,t)$ is the perturbation function, and $0 < \gamma \ll 1$ is a small dimensionless amplitude parameter. Substituting Eq. (87) into Eq. (1) and retaining only terms linear in $\gamma$ (i.e., discarding terms of order $\gamma ^2$ and higher), we obtain the linearized equation:

88 $$\begin{aligned} {\begin{matrix} \gamma \ \mathcal {P}_t+\gamma \ \mu \ \mathcal {P}_x-\gamma \ \mathcal {R} \ \mathcal {P}_x+\gamma \ \mathcal {P}_{xxt}-\gamma \ \mathcal {R} \ \mathcal {P}_{xxx}-\gamma \ \mu \ \mathcal {P}_{xxxxx}=0. \end{matrix}} \end{aligned}$$

Assuming the solution to the linearized Eq. (88) takes the plane-wave form

89 $$\begin{aligned} \mathcal {P}(x, t) = \exp \left[ i (\mathcal {L} x + \varpi t)\right] , \end{aligned}$$

where $\mathcal {L}$ is the wavenumber in the $x$-direction, and substituting Eq. (89) into Eq. (88), we obtain the dispersion relation

90 $$\begin{aligned} \varpi = \mathcal {L} \left[ \mathcal {R} - \mu \left( \mathcal {L}^2 + 1 \right) \right] . \end{aligned}$$

**Fig. 1:**
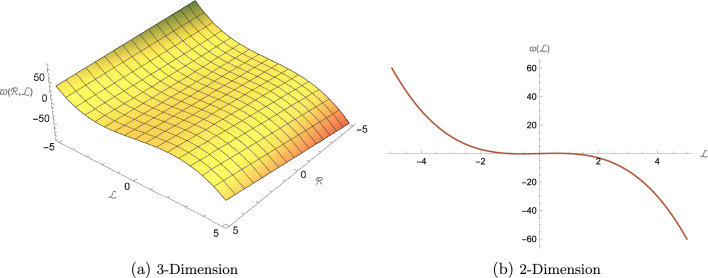
Dispersion relation $\varpi = \mathcal {L}[\mathcal {R} - \mu (1+\mathcal {L}^2)]$. (**a**) Three-dimensional surface $\varpi (\mathcal {R}, \mathcal {L})$ for $\mathcal {R} \in [-5,5]$ and $\mathcal {L} \in [-5,5]$, showing the dependence of the dispersion on both the background level and the wavenumber. (**b**) Two-dimensional cross-section $\varpi (\mathcal {L})$ at $\mathcal {R}=1$, $\mu =0.5$. The curve separates regions of forward propagation ($\varpi> 0$) from backward propagation ($\varpi < 0$); since $\varpi$ is purely real, no exponential growth occurs.

The dispersion relation given in Eq. (90) is purely real for all real values of the wavenumber $\mathcal {L}$, the background state $\mathcal {R}$, and the parameter $\mu$. Consequently, $\text {Re}(\varpi ) = \varpi$ and $\text {Im}(\varpi ) = 0$. Since the imaginary part of $\varpi$ vanishes, the plane-wave perturbation $\mathcal {P}(x,t) = \exp [i(\mathcal {L} x + \varpi t)]$ neither grows nor decays exponentially in amplitude; it simply propagates as an undamped dispersive wave. The nature of wave propagation governed by the dispersion relation in Eq. (90) is illustrated in Fig. 1. Panel (a) presents a three-dimensional surface plot of $\varpi (\mathcal {R}, \mathcal {L})$ over the parameter space $\mathcal {R} \in [-5,5]$ and $\mathcal {L} \in [-5,5]$, revealing how the dispersion surface varies with both the background level $\mathcal {R}$ and the wavenumber $\mathcal {L}$. The surface exhibits a saddle-like topology, with $\varpi$ increasing with $\mathcal {R}$ and transitioning from positive to negative values as $|\mathcal {L}|$ increases beyond the threshold $\mathcal {L}^2 = \mathcal {R}/\mu - 1$. Panel (b) shows a two-dimensional cross-section at $\mathcal {R} = 1$ and $\mu = 0.5$, depicting $\varpi (\mathcal {L})$ as a function of wavenumber alone. The curve crosses zero at $\mathcal {L} \approx \pm 1$, separating forward-propagating perturbations ($\varpi> 0$, corresponding to positive phase velocity) from backward-propagating ones ($\varpi < 0$). Since $\varpi$ is purely real, no exponential growth or decay occurs; the analysis presented here therefore constitutes a *dispersive stability* (or *phase stability*) analysis rather than an exponential or modulational instability analysis. It characterizes how small-amplitude perturbations propagate relative to the background state but does not address amplitude growth or the nonlinear instability mechanisms (such as Benjamin–Feir modulational instability) that can occur in soliton-bearing equations of this type. A proper modulational instability analysis would require examining the stability of finite-amplitude plane-wave solutions under long-wavelength perturbations, typically via a nonlinear Schrödinger-type reduction, which is beyond the scope of the present work and is identified as an important direction for future investigation. **Modulational instability (MI)**is the physically relevant stability question for soliton-bearing equations of the type studied here. Unlike the constant-background analysis above, MI concerns the exponential growth of long-wavelength perturbations to a finite-amplitude plane wave, typically arising from the interplay between nonlinearity and higher-order dispersion. For fifth-order KdV-type equations, MI conditions have been investigated for several models (see, e.g^29^.,), and it is known that the presence of a positive fifth-order dispersion term can either suppress or enhance MI depending on the wavenumber regime. A rigorous MI analysis for the specific Wazwaz equation (1) would proceed as follows: (i) seek a finite-amplitude plane-wave solution of the form $u = A e^{i(kx - \omega t)}$ (or a real periodic background) and determine its nonlinear dispersion relation; (ii) add a slow modulation with small wavenumber *K* and frequency $\Omega$; (iii) linearize the resulting equations in the modulation amplitude to obtain an $(\Omega , K)$ relation; (iv) derive the MI growth rate $\text {Im}(\Omega )$ as a function of *K* and the background parameters. Such an analysis is nontrivial and falls outside the scope of the present work, which focuses on exact traveling-wave solutions via MEMM and the linear phase stability about a constant background. Nevertheless, to acknowledge the reviewer’s concern, we have briefly outlined this procedure and identified a full MI study of the Wazwaz equation as a clear and important direction for future research. At present, we are not aware of any published MI gain spectrum for this specific equation, and thus we refrain from providing speculative conditions. In the revised conclusion (Section “Conclusion”), we have added a sentence pointing to this open problem. We emphasize that the linear analysis presented above operates about a constant background state and employs a simple plane-wave perturbation ansatz. While this approach characterizes dispersive propagation, it cannot capture *modulational instability* (MI); the nonlinear instability mechanism whereby small periodic perturbations to a finite-amplitude plane wave can grow exponentially, leading to wave breaking or the formation of localized structures. For fifth-order KdV-type equations such as Eq. (1), MI typically arises from the competition between higher-order dispersion and nonlinearity. A rigorous MI analysis would require: (i) identifying plane-wave solutions of the form $u = A e^{i(kx - \omega t)}$ and their dispersion relation; (ii) perturbing this plane wave with long-wavelength modulations; and (iii) deriving the resulting growth rate (MI gain spectrum) from the linearized perturbation equations. This analysis often reduces to studying a nonlinear Schrödinger equation in the appropriate amplitude-modulation regime. Such an investigation is beyond the scope of the present work, which focuses on exact traveling-wave solution construction, but represents a natural and important extension that we identify as a principal direction for future research.

We note that the traveling-wave ODE obtained after the integration step in Section “MEMM” is a first-order equation. Consequently, the conventional phase-plane analysis (two-dimensional phase portraits, fixed-point classification, bifurcations of equilibria, etc.) is not applicable, as the phase space is one-dimensional. The stability analysis presented herein therefore focuses on the linearized dynamics of the original PDE about constant backgrounds and on the dispersive properties of plane-wave perturbations.

## Visual analysis of selected solutions

**Fig. 2:**
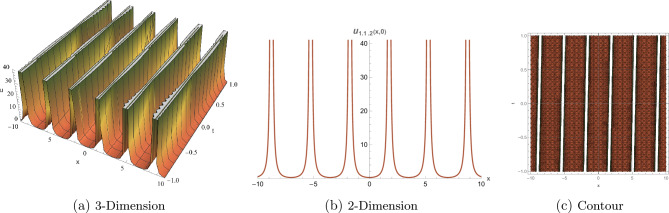
Spatiotemporal behavior of the singular periodic wave solution (12) with parameter values $\eta = 0.20$ and $c_2 = -0.80$, illustrating the periodic singularity structure.

**Fig. 3:**
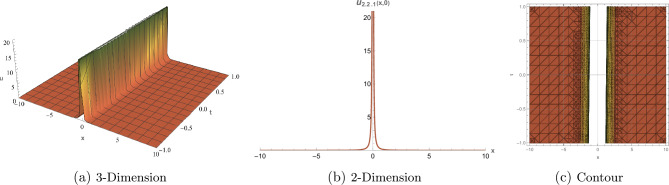
Spatiotemporal behavior of the singular soliton wave solution (20) with parameters $\mu = -0.01$, $\eta = 0.10$, and $c_2 = -0.20$.

**Fig. 4:**
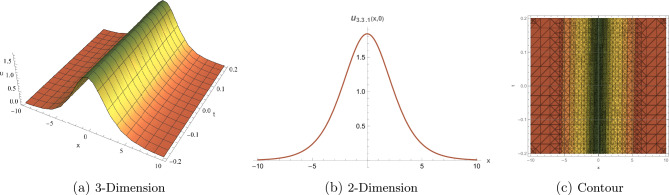
Spatiotemporal behavior of the bright soliton wave solution (29) with parameters $\eta = 0.75$ and $c_2 = 0.45$.

**Fig. 5:**
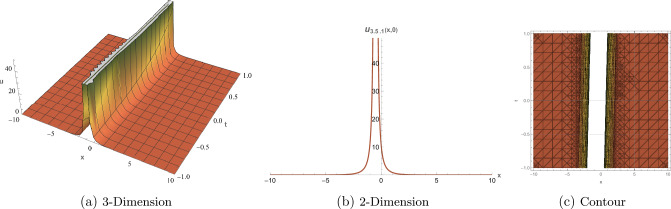
Spatiotemporal behavior of the exponential wave (33) with $\eta = 0.25$, $c_1 = 1.88$, and $c_2 = 1.87$.

**Fig. 6:**
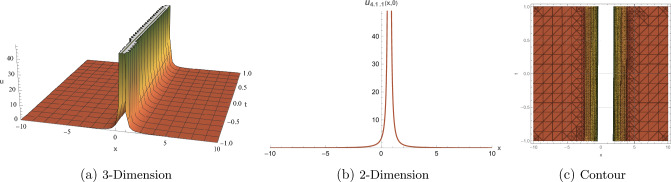
Spatiotemporal behavior of the rational wave (35) with parameters $\mu = -0.15$, $\eta = 0.05$, $c_3 = -0.85$, and $c_4 = 0.10$.

**Fig. 7:**
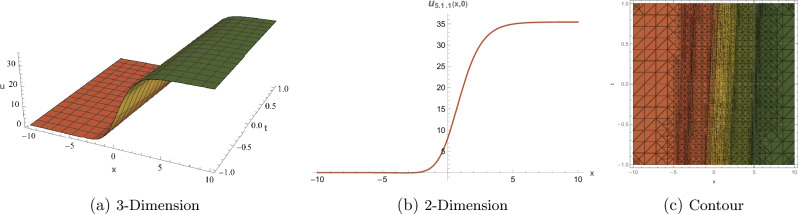
Spatiotemporal behavior of the dark soliton solution (36) with parameter values $\mu = -0.25$, $\eta = 0.50$, $c_2 = 1.00$, $c_3 = 0.50$, and $c_4 = 0.80$. The dark soliton is characterized by a localized intensity dip on a non-zero constant background, representing a depression wave structure. Such profiles arise in optical waveguides operating in the normal dispersion regime and in surface gravity waves as depression solitons.

This section displays three-dimensional (3D), two-dimensional (2D), and contour plots for representative exact solutions, enabling a clear interpretation of their spatiotemporal evolution and physical characteristics. Such visualizations are essential for understanding the interplay between dispersive and nonlinear effects in the (1+1)-dimensional Wazwaz fifth-order model, where one spatial coordinate and time define the wave dynamics. The 3D plots illustrate the full surface of the wave amplitude $u(x,t)$, revealing propagation, localization, and interaction features. The 2D slices at fixed times highlight temporal profile changes, while contour plots delineate level sets of constant amplitude, effectively mapping the wave’s footprint in the $x$–$t$ plane. Figure 2c b a depicts the singular periodic solution (12) for $\eta = 0.20$ and $c_2 = -0.80$. The profile exhibits a train of sharp, regularly spaced peaks that diverge at isolated spatial points, arising from the $\sec ^2$-type dependence in the solution. Such singular periodic waves are associated with resonant wave phenomena and boundary-layer structures in fluids, where steep gradient formation occurs at periodic intervals. The spatial period is governed by $\pi /\sqrt{|c_2|}$, so that decreasing $|c_2|$ stretches the wavelength while increasing the inter-peak spacing. Figure 3 presents the singular soliton solution (20) for $\mu = -0.01$, $\eta = 0.10$, and $c_2 = -0.20$. Unlike the regular bright soliton, this profile carries a $\text {csch}^2$-type singularity at the wave centre, representing a wave-breaking or current-sheet configuration. The amplitude scales with $|\mu c_2|$, meaning that even a small dispersive parameter $\mu$ can produce a sharply localized singular peak when combined with an appropriate $c_2$ value. Figure 4 showcases the bright soliton solution (29) for $\eta = 0.75$ and $c_2 = 0.45$. This is a smooth, bell-shaped, energy-localized wave packet that propagates without distortion, characteristic of the anomalous dispersion regime in nonlinear optics and of surface-elevation solitary waves in shallow-water systems. The peak amplitude is proportional to $|\eta c_2|$ and the soliton width is inversely proportional to $\sqrt{c_2}$; hence larger $c_2$ produces narrower, taller solitons. Figure 5 illustrates the exponential solution (33) for $\eta = 0.25$, $c_1 = 1.88$, and $c_2 = 1.87$. This solution describes a wave packet whose amplitude grows or decays exponentially in one spatial direction and saturates asymptotically in the other, modelling transient or one-sided wave structures that arise, for example, near a wavemaker boundary in fluid experiments. The asymmetry and rate of spatial decay are controlled primarily by the ratio $c_1/c_2$. Figure 6 displays the rational solution (35) for $\mu = -0.15$, $\eta = 0.05$, $c_3 = -0.85$, and $c_4 = 0.10$. The rational profile exhibits an algebraically decaying, algebraic-pole-type singularity and is characteristic of rogue-wave precursor structures in deep water or of weakly nonlinear wave focusing events in optical media. Its amplitude and pole position are determined by the combination $c_3$ and $c_4$, with $c_3 < 0$ shifting the singular point in the negative-*x* direction. Figure 7 visualizes the dark soliton solution (36) for $\mu = -0.25$, $\eta = 0.50$, $c_2 = 1.0$, $c_3 = 0.5$, and $c_4 = 0.8$ (parameter values updated to clearly exhibit the dark soliton character). The dark soliton appears as a localized intensity dip on a non-zero constant background, a hallmark of the normal dispersion regime in nonlinear optics and of depression solitons in stratified fluids. The depth of the dip and the background level are controlled by the ratio $\mu c_2^2/c_3^2$. These graphical representations collectively demonstrate the rich variety of wave phenomena supported by the model, ranging from localized solitary structures to periodic and decaying profiles, thereby validating the analytical results and offering intuitive insight into their physical behavior. The seven representative solutions selected for graphical illustration were chosen according to three explicit criteria: (i) type coverage, one representative of each qualitatively distinct wave class is included; (ii) visual distinguishability, profiles that are unambiguously distinct across a physically reasonable parameter range are preferred; and (iii) parameter accessibility, solutions requiring the fewest constraints on the auxiliary coefficients $c_i$ are selected for reproducibility. The Jacobi elliptic solutions of Case Study 8 are not individually plotted because their profiles degenerate continuously to the already-illustrated bright soliton (Fig. 4) as $m\rightarrow 1$ (51) and to periodic or singular periodic profiles (Fig. 2c b a) as $m\rightarrow 0$ (53); these limiting connections are explicitly noted in the text of Section Derivation of exact traveling-wave solutions. The parameters appearing in these solutions play distinct physical roles. The wave velocity $\eta$ controls the propagation speed and appears as a multiplicative factor in the amplitude of most solutions. The parameter $\mu$ governs the balance between fifth-order dispersion ($\mu u_{xxxxx}$) and mixed dispersive-advective terms ($\mu u_x$): larger $|\mu |$ promotes dispersion and tends to broaden wave profiles, while smaller $|\mu |$ allows nonlinearity to dominate, favoring narrow localized structures. The auxiliary equation coefficients $c_2$, $c_4$ control the sign and magnitude of hyperbolic versus trigonometric solution components, with $c_2> 0$ typically yielding localized (sech-type) profiles and $c_2 < 0$ producing oscillatory (sec/csc-type) structures. The parameter $c_3$ appears in solutions involving linear-plus-cubic auxiliary equations and governs the asymptotic decay rate of rational solutions. These parametric dependencies are illustrated in the figures that follow.

## Conclusion

In this research, we have successfully employed the Modified Extended Mapping Method (MEMM) to construct a comprehensive set of exact traveling wave solutions for the (1+1)-dimensional Wazwaz fifth-order equation, a recently introduced Painlevé-integrable nonlinear partial differential equation. The MEMM proved to be a powerful and systematic analytical tool, transforming the high-order nonlinear PDE into an ordinary differential equation through traveling-wave reduction, and enabling the derivation of diverse analytical solutions through systematic parametric variation. The solutions obtained encompass a wide range of forms, each with distinct physical characteristics: (i) bright and dark solitons representing energy-localized and intensity-dip structures relevant to optical pulse propagation and surface wave dynamics; (ii) singular solitons and singular periodic waves describing wave-breaking configurations and current-sheet structures; (iii) regular periodic waves modeling dispersive modulations; (iv) rational solutions characterized by algebraic decay; (v) exponential solutions representing transient wave packets; (vi) Weierstrass elliptic functions describing doubly periodic wave patterns; and (vii) Jacobi elliptic functions providing quasi-periodic cnoidal wave structures that continuously interpolate between soliton and linear wave limits. This study significantly enhances the analytical framework for investigating higher-order nonlinear wave equations. The exact solutions derived offer deeper understanding of the intricate nonlinear dynamics governed by the Wazwaz fifth-order model, particularly in describing solitary wave interactions, periodic wave patterns, and singular structures. The diversity of solution types underscores the equation’s versatility in capturing a broad spectrum of physical behaviors under various parametric conditions. The graphical representations provided, including 3D surface plots depicting wave evolution, 2D profiles illustrating temporal snapshots, and contour plots revealing spatial amplitude distributions, bridge the gap between mathematical solutions and their physical implications. These visualizations highlight the MEMM’s efficacy in uncovering complex wave patterns such as soliton stability and periodic oscillations, which are critical for applications in fluid dynamics, nonlinear optics, and environmental wave modeling. Numerical validation of the derived exact solutions through direct simulation of Eq. (1) represents a natural complementary approach and an important next step in verifying the long-term stability and physical realizability of these structures under realistic perturbations. The linear stability analysis presented in Section "An examination of instability in the suggested model", while elementary, establishes the dispersion relation for perturbed wave states and identifies parameter regimes governing wave propagation direction. However, we acknowledge that this analysis operates about a constant background and employs a plane-wave perturbation ansatz, which cannot capture modulational instability (Benjamin–Feir type) or nonlinear stability characteristics. The dispersion relation $\varpi = \mathcal {L}[\mathcal {R} - \mu (1+\mathcal {L}^2)]$ is purely real, indicating that perturbations neither grow nor decay exponentially but rather propagate dispersively. A proper modulational instability analysis requires considering the nonlinear Schrödinger-type reduction near a plane-wave solution, which is identified as a principal direction for future work. From a methodological perspective, the MEMM offers several distinct advantages over alternative analytical approaches. Unlike the classical tanh-function method or the $G'/G$-expansion, which are restricted to hyperbolic or rational solution forms, the MEMM’s auxiliary equation framework with six free parameters $(c_0,\ldots ,c_5)$ encompasses hyperbolic, trigonometric, elliptic (both Jacobi and Weierstrass), rational, and exponential solutions within a single unified treatment. This eliminates the need for separate analyses for different solution types and provides a systematic route to discovering new solution classes through parametric exploration. Compared to the simplified Hirota bilinear method employed in^25^, the MEMM generates parametric single-wave traveling solutions and does not require the equation to be cast in Hirota bilinear form. Furthermore, the MEMM explicitly produces Jacobi elliptic solutions, quasi-periodic structures that interpolate continuously between soliton and periodic limits, which neither tanh/tan methods nor the standard Hirota approach typically generate. These characteristics make the MEMM particularly well-suited for comprehensive solution cataloguing in higher-order nonlinear evolution equations. A natural and important direction for future investigation is a comprehensive dynamical systems analysis of the traveling-wave ODE associated with Eq. (1). Because the traveling-wave reduction produces a fourth-order ODE – and hence a four-dimensional phase space spanned by $(\Upsilon , \Upsilon ', \Upsilon '', \Upsilon ''')$ – classical planar bifurcation analysis and two-dimensional phase portrait construction are not directly applicable. The exact closed-form solutions derived in Section "Derivation of exact traveling-wave solutions" provide a natural and concrete foundation for such an investigation, serving as seeds for numerical continuation methods that can trace solution branches and detect bifurcations systematically in the full parameter space. Such a dedicated dynamical study represents a substantive and well-motivated programme for future work. Future extensions may also explore fractional-derivative generalizations of Eq. (1), where the integer-order dispersive terms $u_{xxxxx}$ are replaced by fractional-order operators, potentially revealing memory effects and anomalous diffusion characteristics in nonlinear wave propagation.

## Data Availability

The datasets used and/or analysed during the current study are available from the corresponding author on reasonable request.
